# Therapeutic Potential of Tricyclic Pyridazinone-Based Molecules: An Overview

**DOI:** 10.3390/ijms26083806

**Published:** 2025-04-17

**Authors:** Battistina Asproni, Gérard A. Pinna, Paola Corona, Silvia Coinu, Sandra Piras, Antonio Carta, Gabriele Murineddu

**Affiliations:** Department of Medicine, Surgery and Pharmacy, University of Sassari, Via Muroni 23/A, 07100 Sassari, Italy; pinger@uniss.it (G.A.P.); pcorona@uniss.it (P.C.); s.coinu1@phd.uniss.it (S.C.); piras@uniss.it (S.P.); acarta@uniss.it (A.C.)

**Keywords:** heterocyclic, tricyclic pyridazin-3(2*H*)one, anti-inflammatory, antiulcer, anticancer

## Abstract

Pyridazin-3(2*H*)one-based molecules have always attracted the attention of medicinal chemists due to their different pharmacological properties. The incorporation of such nuclei in therapeutically active molecules either as monocyclic units or as fused bi- or tricyclic scaffolds results in a wide range of pharmacological effects such as anti-inflammatory, analgesic, anticancer, antimicrobial, antiviral, cardiovascular-protective, antiulcer, and many other useful pharmacological activities. In accordance with our consolidated experience gained over the years in the chemistry and biology of tricyclic pyridazin-3(2*H*)ones, this review summarizes SAR studies of such pyridazinone-based polycyclic compounds endowed with various biological and therapeutic properties.

## 1. Introduction

**Figure 1:**
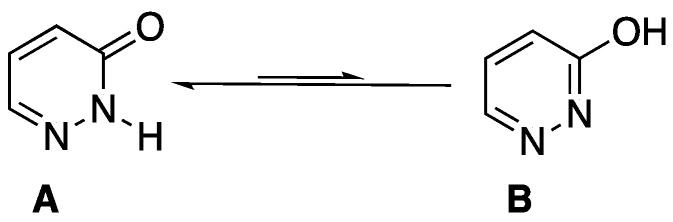
Keto-enol tautomerism of pyridazine-3(2*H*)one.

Heterocyclic compounds, particularly those containing one or more nitrogen atoms, have long attracted interest due to their crucial role in drug discovery. These molecules can function as pharmacologically active entities alone, with various substituents, or they can be fused with other (hetero)cyclic systems to form linear or, more often, angular polycyclic structures. This duality has made them widely studied and applicable in a wide range of pharmacological effects [1,2]. Pyridazine (1,2-diazine) is a heterocyclic nucleus derived from the replacement of two carbon atoms in the benzene ring with the same number of nitrogen atoms. Among its oxygen-substituted analogs, pyridazin-3(2*H*)one **A** is a more stable oxo-form of 3-hydroxyl pyridazine **B** (Figure 1).

The pyridazine-3(2*H*)one nucleus is known as a privileged scaffold and also as the wonder nucleus because of its numerous applications in different areas, especially as a pharmacophoric element in therapeutically active drugs. Structurally, the presence of nitrogen atoms and the keto functionality can provide protonation reactions, as well as hydrogen bond formation, which can account for the variety of pharmacological properties of small molecules containing a pyridazinone ring [3,4].

A survey of the literature revealed several hundred articles encompassing appropriate substituted pyridazine-3(2*H*)one-based molecules or structures in which this nucleus appears in fused bicyclic scaffolds. Furthermore, the analysis of these publications highlighted more sophisticated structures incorporating this nucleus in tricyclic or even tetracyclic molecular architectures endowed with a broad range of biological activities. Pyridazine-3(2*H*)one-based molecules, both saturated and unsaturated, have been reported to have diverse pharmacological activities such as anti-inflammatory, analgesic, anticancer, antimicrobial, antiviral, cardiovascular-protective, antiulcer, and many other useful pharmacological activities [3,4,5,6].

**Scheme 1:**
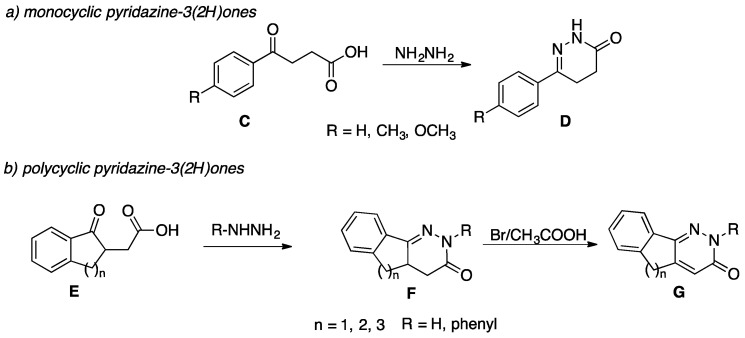
Synthesis of 6-substituted-phenyl-4,5-dihydropyridazin-3(2*H*)-ones **D** and benzocycloalkylpyridazine-3(2*H*)ones **F** and **G**.

Several synthetic strategies were described in the literature for the synthesis of pyridazine-3(2*H*)-one-based molecules [7]. A simple method for the synthesis of monocyclic 6-substituted-phenyl-4,5-dihydropyridazin-3(2*H*)-ones (Scheme 1, a, **D**) involved the addition of hydrazine hydrate to the appropriate γ-ketoacid (**C**) [8]. The addition of hydrazine or even substituted hydrazine to appropriate bicyclic γ-ketoacid **E** (b) provided several tricyclic benzocycloalkylpyridazine-3(2*H*)ones **F** in high yields, which were oxidized efficiently with bromine in acetic acid to the corresponding pyridazinones **G** [9,10].

**Figure 2:**
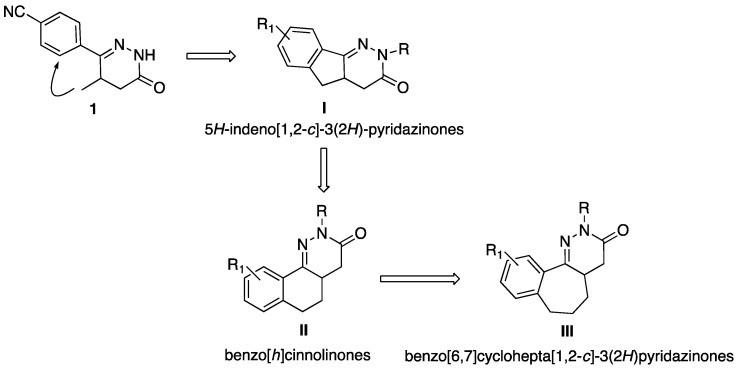
Design of benzocycloalkylpyridazinone-based tricyclic scaffolds **I**–**III**.

Our interest in tricyclic pyridazine-3(2*H*)one derivatives dates back several decades, when a series of benzocycloalkylpyridazinone-based tricyclic scaffolds of general formula **I**–**III** (Figure 2) were designed as rigid analogs of 5-methyl-6-*para*-cyanophenyl-4,5-dihydro-3(2*H*)-pyridazinone **1**, endowed with antihypertensive activity together with inotropic, vasodilator, and antiaggregating properties. It was observed that such pharmacological effects are at least partly mediated by the inhibition of phosphodiesterase III (PDEIII) [11].

It has been suggested that minimization of the flexibility of the lead compound **1** through the introduction of structural constraints could have some effect on the biological activity and is likely to allow the ligand to bind to its biological target with a high degree of affinity and selectivity. Modifications carried out on such scaffolds **I**–**III**, i.e., dehydrogenation, isomerization, and bioisosterism and their fine tuning (i.e., by varying R and R_1_ substituents), allowed the identification of hundreds of molecules, most of them endowed with different pharmacological properties (vide infra). Herein, we briefly present the bioactivities and SAR studies of pyridazinone-based tricyclic or even tetracyclic compounds, most of which are related to **I**–**III** scaffolds, that have been investigated by us and other groups. In addition, this review encompasses the biological literature of some other most relevant tricyclic-based molecules that are quite different from **I**–**III**, featuring the pyridazine-3(2*H*)one core in their structure.

## 2. Anti-Inflammatory/Analgesic/Antipyretic Activities

Inflammation is part of the biological response of the body’s tissues to harmful stimuli, such as pathogens, damaged cells, or irritants. Chronic or uncontrolled inflammation plays an important role in the initiation and development of diseases such as rheumatoid arthritis, heart disease, cancer, multiple sclerosis, diabetes, gout, psoriasis, aging, and neurodegenerative diseases [12,13]. Non-steroidal anti-inflammatory drugs, opioids, and corticosteroids are widely used in clinical practice to treat inflammatory and painful conditions, although they are associated with several side effects [14]. A survey of the recent literature revealed pyridazine/pyridazine-3(2*H*)one as very appealing scaffolds for anti-inflammatory and analgesic drug discovery [3,4,15].

**Figure 3:**
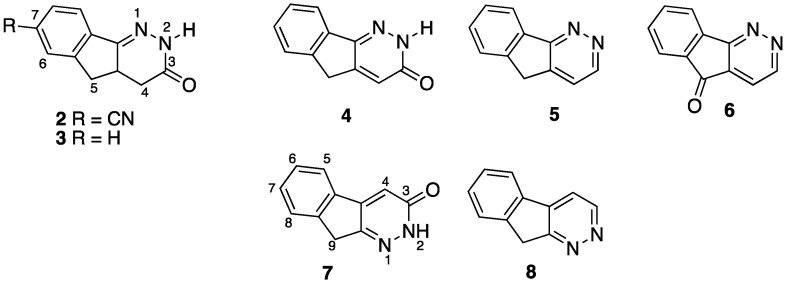
Structures of indeno-pyridazinones **4** and **7** and indeno-pyridazines **5**, **6**, and **8**.

The discovery of tricyclic pyridazine-3(2*H*)one derivatives as anti-inflammatory agents started in our laboratories as a part of a structure–activity relationship (SAR) study carried out by Cignarella et al., allowing the serendipitous identification of 7-cyano-4,4*a*-dihydro-5*H*-indeno[1,2-*c*]-3(2*H*)-pyridazinone **2** (Figure 2 and Figure 3) endowed with anti-inflammatory properties. This derivative lacked any detectable antihypertensive activity compared to the lead compound **1**, while being active as an anti-inflammatory agent, along with **3**, in a carrageenin-induced rat paw edema model, with activity comparable to that of acetylsalicylic acid (ASA, Table 1) [16]. Dehydrogenation and full aromatization of 4,4*a*-dihydro-5*H*-indeno[1,2-*c*]-3(2*H*)-pyridazinone **3** gave rise to 5*H*-indeno[1,2-*c*]-3(2*H*)-pyridazinone **4** and 5*H*-indeno[1,2-*c*]pyridazine **5**, respectively, both endowed with anti-inflammatory activity. Oxidation of the methylene group at the C-5 position of compound **5** to ketone **6**, as well as isomerization of compounds **4** and **5** to give 9*H*-indeno[2,1-*c*]-3(2*H*)-pyridazinone **7** and 9*H*-indeno[2,1-*c*]pyridazine **8**, respectively, kept the anti-inflammatory activity comparable to ASA. These compounds, and their related analogs, exhibited analgesic and antipyretic properties to various extents [17,18].

## 3. Cardiovascular Activity

It is well established that cardiovascular disease, in particular coronary heart disease and stroke, as well as peripheral arterial disease and aortic disease, is the leading cause of mortality in modern societies [19,20]. Hypertension is a chronic medical condition in which the arterial blood pressure is persistently elevated and contributes to increased risk of heart attack, stroke, heart failure, and other complications, along with raised blood glucose, raised blood lipids, and obesity. Several pyridazinone-based compounds exhibit antihypertensive activity [21,22]. The molecular mechanisms mediating this effect may ultimately involve a decrease in intracellular calcium concentrations.

**Figure 4:**
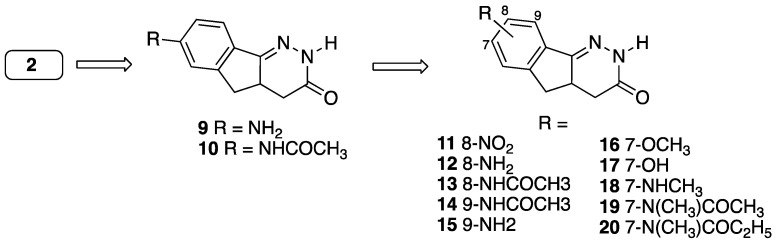
Structure of 5*H*-indeno[1,2-*c*]-3(2*H*)-pyridazinones **9**–**20**.

As part of our ongoing efforts to generate SARs around 7-cyano-4,4*a*-dihydro-5*H*-indeno[1,2-*c*]-3(2*H*)-pyridazinone **2** (Figure 3), it was found that when the cyano group was replaced by amino or acetylamino groups, the resulting compounds **9** and **10** (Figure 4) showed a potent blood-pressure-lowering effect (reduction of 20–50 mmHg) on spontaneously hypertensive rats (SHRs), unlike the reference derivative **2** which displayed only anti-inflammatory activity [23]. A SAR study on the antihypertensive activity of 4,4*a*-dihydro-5*H*-indeno[1,2-*c*]-3(2*H*)-pyridazinone-based molecules identified derivatives **18**–**20** (Figure 4), which had antihypertensive activity comparable to **9** and **10**, even if with a short duration of action [24]. Furthermore, compounds **9** and **10**, like most analogs **11**–**20**, were also active as antithrombotic agents, evaluated in a mouse model as percent protection against death or paralysis of the hind limbs induced by a thrombotic mixture (collagen and adrenalin), with an activity comparable to or higher than that of ASA. In addition, compounds **9** and **10** exhibited anti-inflammatory activity and platelet aggregation-inhibiting activity, evaluated using guinea pig platelet-rich plasma and inducing aggregation by either ADP, collagen, or thrombin, with derivative **10** also showing potent antiulcer properties in a rat indomethacin-induced ulcer model (vide infra) [23].

**Figure 5:**
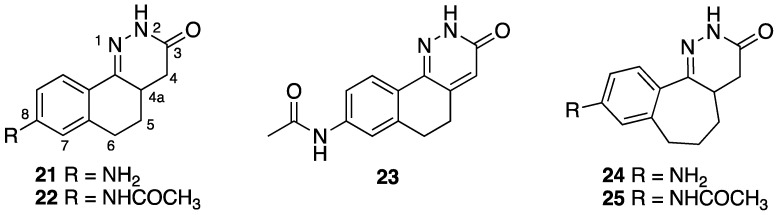
Structure of 4,4*a*,5,6-tetrahydrobenzo[*h*]cinnolinones **21** and **22**, 5,6-dihydrobenzo[*h*]cinnolinone **23**, and benzo[6,7]cyclohepta[1,2-*c*]-3(2*H*)-pyridazinones **24** and **25**.

Within this framework, several substituted benzo[*h*]cinnolinones and benzo[6,7]cyclohepta[1,2-*c*]-3(2*H*)-pyridazinones of general formula **II** and **III** (Figure 2) were designed as higher homologues of **I** in order to check whether the planarity of the structure **I** is in line with the maximum activity observed in derivatives previously described. These homologues were tested for antihypertensive, inotropic, antithrombotic, anti-inflammatory, and antiulcer activity. While the seven-membered ring compounds **24** and **25** (Figure 5) exhibited only antithrombotic properties comparable to ASA, most of the benzo[*h*]cinnolinones **21**–**23** displayed significant antihypertensive, inotropic, and antithrombotic properties. Furthermore, the D-isomer of **22** was more active than the racemic form in lowering blood pressure, with less tachycardic effects. Moreover, unlike the 5*H*-indeno[1,2-*c*]-3(2*H*)-pyridazinone series (Figure 4), these derivatives were devoid of anti-inflammatory or antiulcer activity [25].

**Figure 6:**
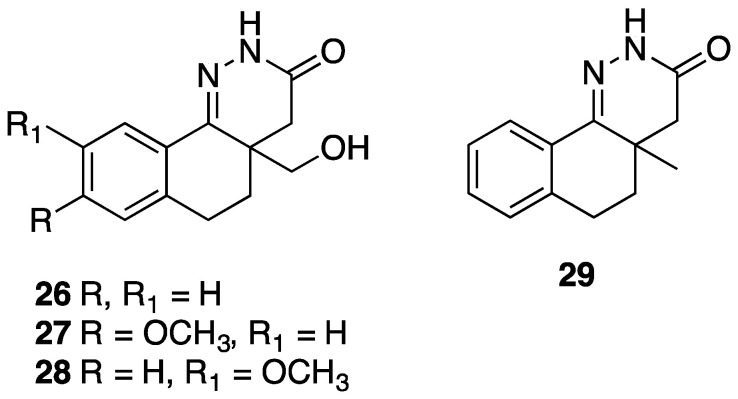
Structure of 4,4*a*,5,6-tetrahydro-4*a*-substituted-benzo[*h*]cinnolinones **26**–**29**.

Several 4,4*a*,5,6-tetrahydro-4*a*-substituted-benzo[*h*]cinnolinones were synthesized and evaluated for antihypertensive and antithrombotic activity compared to the 4*a*-unsubstituted congeners. Most of the tested compounds displayed significant antihypertensive activity. In particular, compound **26** (Figure 6) was more active than the 4*a*-unsubstituted analog, whereas compounds **27**–**29** showed antithrombotic activity comparable to or greater than that of ASA [26].

Warrington et al. reported the cardiotonic properties (inotropic and vasodilator activity) of a series of 4,4*a*-dihydro-5*H*-indeno[1,2-*c*]-3(2*H*)-pyridazinone-based molecules, including derivative **10** (Figure 4), and of benzo[*h*]cinnolinones, including compound **22** (Figure 5). The authors correlated the observed activity mainly with the ability of such compounds to inhibit PDEIII [11].

Some phenyl-4,5-dihydro-3(2*H*)-pyridazinones related to compound **1** (Figure 2), tricyclic indenopyridazinones **3**, **9**, and **10**, as well as benzocinnolinones **21** and **22** and benzocycloheptapyridazinones **24** and **25** were submitted for conformational analysis to better define the relationship between their cardiovascular properties and their preferred conformation. Analogs of compound **1**, indenopyridazinone and benzocinnolinone derivatives, which give rise to highly active compounds, were found to exist mainly in a conformation with a near-planar arrangement of the phenyl and pyridazinone core. Compounds belonging to the benzocycloheptapyridazinone series, whose derivatives were generally inactive, showed conformations that deviated from planarity [27].

**Figure 7:**
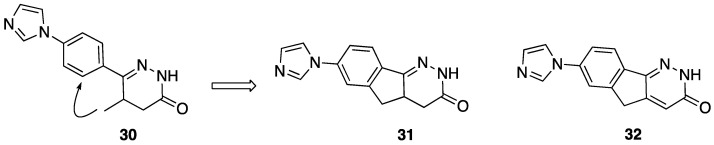
Design of imidazole-based indeno[1,2-*c*]-pyridazinones **31** and **32**.

Taking into account the potent positive inotropic activity of a series of 4,5-dihydro-6-[4-(1*H*-imidazol-1-yl)phenyl]-3(2*H*)-pyridazinones, i.e., compound **30** (Figure 7), Sircal et al. synthesized and evaluated the positive inotropic activity of a series of 5*H*-indeno[1,2-*c*]-3(2*H*)-pyridazinones (including **9**, **10**, Figure 4) and the benzo[*h*]cinnolinone **22** (Figure 5) [28]. Most of these tricyclic pyridazinones (i.e., **31** and **32**) demonstrated strong positive inotropic activity in the same range as that of their freely rotating acyclic analogs. Interestingly, in their screening, compound **10** retained an inotropic activity comparable to or slightly higher than that of the imidazole **31**. By SAR studies, the authors concluded that a generally planar ring structure is desirable for maximum positive inotropic effects. Several of these tricyclic pyridazinones were evaluated for PDEIII-inhibitory activity. Compounds **31** and **32** possessed similar potency (**31**: IC_50_ 1.8 μM, **32**: IC_50_ 1.6 μM) to **30** (IC_50_ 1 μM), which also explained the similar inotropic activity of these compounds.

**Figure 8:**
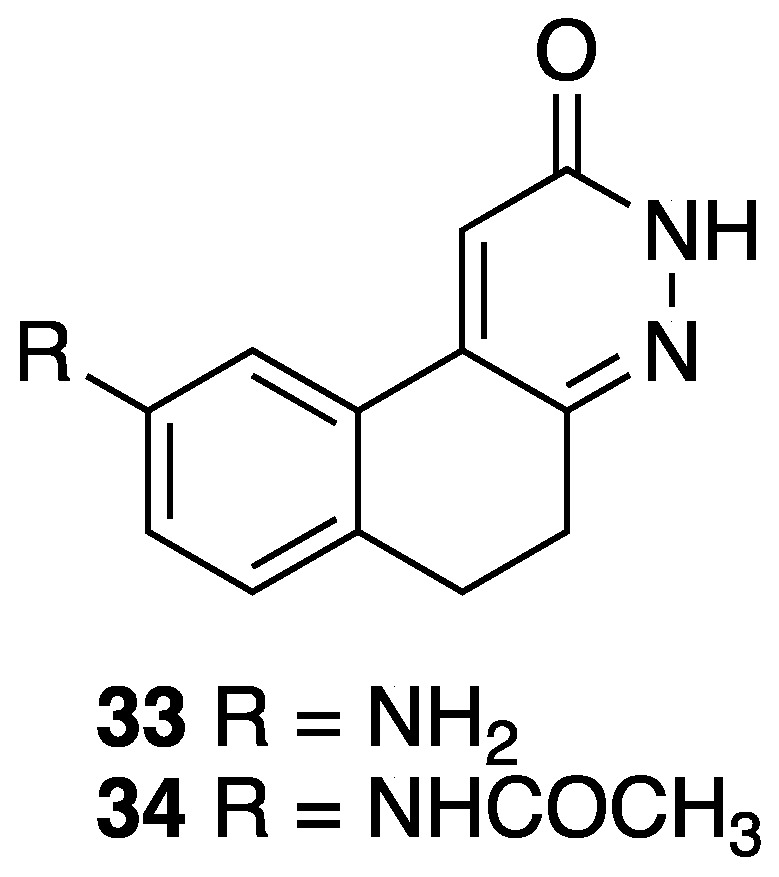
Structure of 5,6-dihydrobenzo[*f*]cinnolin-2(3*H*)ones **33** and **34**.

Our group, by an isomerization approach, designed and synthesized two dihydrobenzo[*f*]cinnolin-2(3*H*)ones **33** and **34** (Figure 8). In comparison with isomeric derivatives **21** and **22** (Figure 5), these compounds were tested for their antihypertensive and antiaggregating activities. In vivo tests showed that such compounds revealed antihypertensive properties weaker than the model compounds. On the contrary, in inhibiting collagen-induced platelet aggregation, both compounds were more potent than **21** and **22** [29].

**Figure 9:**
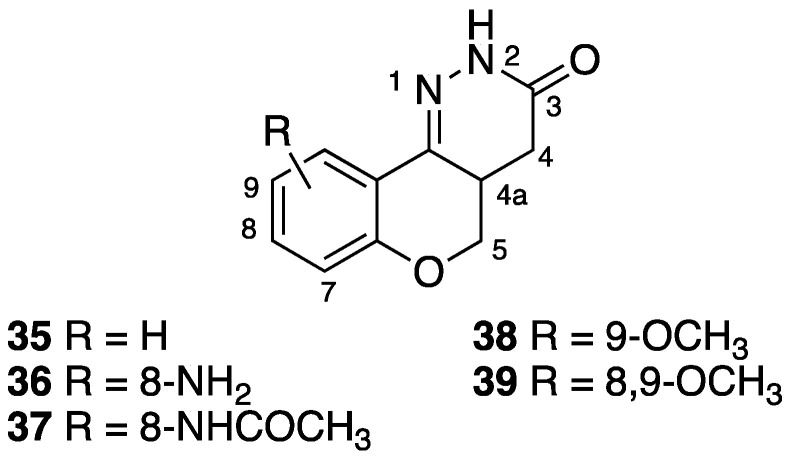
Structure of 4,4a-dihydro-5*H*-[1]benzopyrano[4,3-*c*]pyridazin-3(2*H*)ones **35**–**39**.

Bioisosterism is a useful strategy for the rational design of new drugs [30,31]. Within this framework, our group envisioned that bioisosteric modification of 4,4*a*,5,6-tetrahydrobenzo[*h*]cinnolinone core **II** (Figure 2) might offer new templates that could provide better pharmacological properties. Following this approach, a series of 4,4a-dihydro-5*H*-[1]benzopyrano[4,3-*c*]pyridazin-3(2*H*)ones were designed, synthesized, and tested for their cardiovascular-related properties. The most interesting compounds of this series are depicted in Figure 9. All derivatives were ineffective in lowering the blood pressure in spontaneously hypertensive rats, with the exception of **37**, showing a short-lasting action. Derivatives **36** and **37** were found to be very active as antithrombotics in mice, more so than ASA [32]. Interestingly, many derivatives of this class showed antiulcer activity (vide infra).

**Figure 10:**
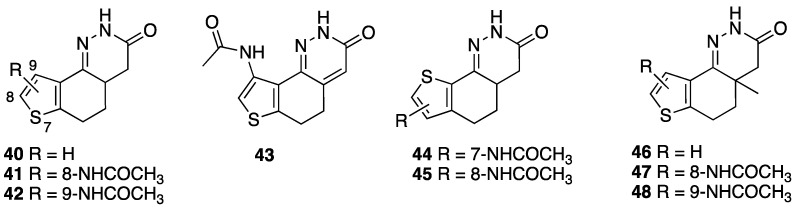
Structure of thieno[2,3-*h*]cinnolin-3(2*H*)-ones **40**–**43** and **46**–**48** and thieno[3,2-*h*]cinnolin-3(2*H*)-ones **44** and **45**.

Following the above-mentioned bioisosteric approach, and taking the significant antihypertensive and antithrombotic properties of 4,4*a*,5,6-tetrahydrobenzo[*h*]cinnolinone derivative **22** (Figure 5) as a reference, the thieno[2,3-*h*]cinnolin-3(2*H*)-ones, **40** and **41** (Figure 10), were designed, synthesized, and pharmacologically evaluated. Compound **41** and the unsubstituted term **40**, with respect to the bioisoster **22**, retained antithrombotic properties which were higher than (**41**) or comparable to those of ASA but without significant antihypertensive activity. Conformational analysis suggested that the absence of activity may result from a different orientation of the acetylamino group in compounds **22** and **41**. Further, thieno[2,3-*h*]- and thieno[3,2-*h*]cinnolin-3(2*H*)-ones, **42**, **43** and **44**, **45**, respectively (Figure 10), were designed, synthesized, and evaluated for their pharmacological profile. In vivo tests revealed that only **45** exhibited antihypertensive properties comparable to compound **22**, while all derivatives had a lower hypotensive activity. All compounds, except **42**, were more potent than **22** in the inhibition of collagen-induced platelet aggregation [33]. The 4*a*-methyl-substituted-thieno[2,3-*h*]cinnolin-3(2*H*)-ones **46**–**48** were also synthesized. Their pharmacological profile was evaluated in comparison to **22**. In vivo tests showed that **46** displayed lower levels of antihypertensive activity with respect to **22**. Tricyclic thienocinnolinones **46**–**48** demonstrated potent platelet-antiaggregating activity [34].

**Figure 11:**
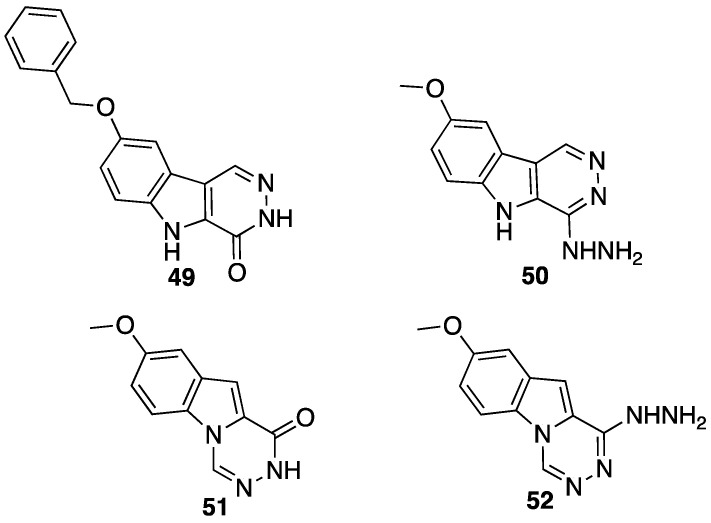
Structure of pyridazino[4,5-*b*]indoles **49**, **50** and 1,2,4-triazino[4,5-*a*]indoles **51**, **52**.

Selective inhibition of thromboxane (TXA_2_) synthetase may be an effective approach for antithrombotic drugs that act on platelet aggregation. TXA_2_, rapidly hydrolyzed to TXB_2_ under physiological conditions, is a potent vasoconstrictor and platelet-aggregating agent. Monge et al. synthesized a series of tricyclic pyridazinone/pyridazine indole-based compounds (**49**–**52**, Figure 11) and tested them for TXA_2_ synthetase inhibitor activity. While compounds **51** and **52** had no significant activity, derivatives **49** and **50** were inhibitors of platelet aggregation induced by ADP in vitro, and both compounds exhibited potent TXA_2_ synthetase inhibition. Compound **50** also showed antihypertensive activity similar to that of hydralazine, but its hydrochloride salt had an acute toxicity in mice about 2.2 times lower than that of hydralazine [35].

The cardiovascular-related properties of most tricyclic pyridazinones described above are summarized in Table 2.

## 4. Antiulcer Activity

**Figure 12:**
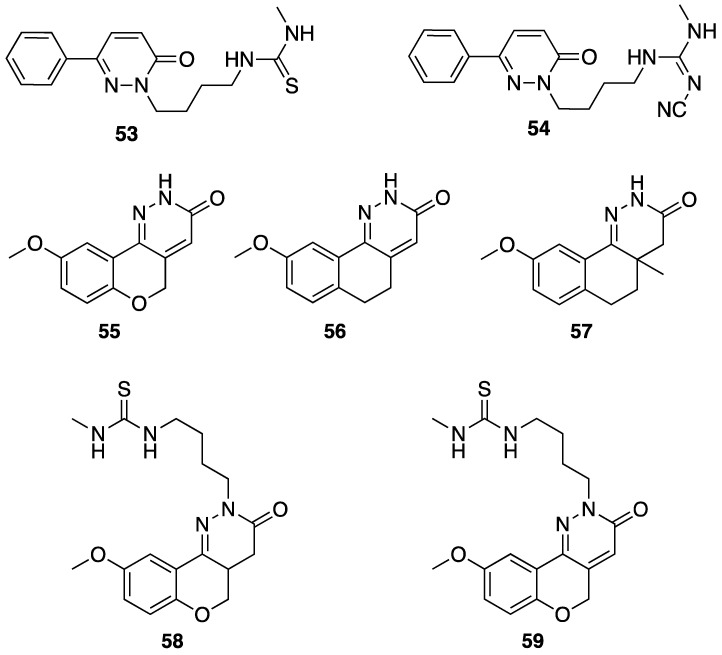
Structure of thiourea or 2-cyanoguanidine-based 3(2*H*)-pyridazinone derivatives **53** and **54**, 5*H*-[1]benzopyrano[4,3-*c*]pyridazin-3(2*H*)ones **55**, **58**, and **59**, and benzo[*h*]cinnolinones **56** and **57**.

Peptic ulcer disease includes gastric or duodenal ulceration, which is a break in the epithelium of the gastric or duodenal mucosa. The most common causes of peptic ulcer disease are the use of non-steroidal anti-inflammatory drugs and *Helicobacter pylori* infection. Management of peptic ulcer disease makes use of antiacids, gastroprotective complexes and chelators, H_2_-receptor antagonists, synthetic prostaglandins, and H^+^/K^+^-ATPase proton pump inhibitors [14]. Despite the wide pharmacological activities of 3-(2*H*)-pyridazinone-based molecules, only a few compounds with gastric antisecretory and/or antiulcer properties have been described. In this context, a series of monocyclic 3(2*H*)-pyridazinone derivatives with a thiourea or cyanoguanidine moiety (**53** and **54**, Figure 12) showed marked gastric antisecretory activity in rats without histamine H_2_-receptor antagonistic action [36].

During the synthesis and pharmacological evaluation of 4,4*a*-dihydro-5*H*-[1]benzopyrano[4,3-*c*]pyridazin-3(2*H*)ones **35**–**39** (Figure 9) and bioisosteres of antihypertensive and antithrombotic benzo[*h*]cinnolinones (**21** and **22**, Figure 5), our group identified compound **38** as a potent antiulcer agent, able to protect rats from the formation of ulcers induced by ASA or phenylbutazone (PBZ) (ED_50_ = 12.2 mg/Kg and 25.4 mg/Kg po in ASA and PBZ models, respectively). In vitro experiments carried out on compound **38** indicated that it has no anticholinergic activity at a concentration of 1 × 10^−5^ M and that it has no histamine H_2_-receptor antagonistic activity at the same concentration [32]. Further SAR studies carried out on 5*H*-[1]benzopyrano[4,3-*c*]pyridazin-3(2*H*)one and benzo[*h*]cinnolinone scaffolds identified further tricyclic 3-(2*H*)-pyridazinone derivatives whose structures are depicted in Figure 12. All compounds **55**–**59** showed statistically significant antiulcer properties in the ethanol model, with **55** being the most active compound. Conversely, only **55** and **59** were found to be weakly active in the ASA model. In a pylorus-ligated rat model, **55** displayed significant antisecretory activity, being able to reduce the acidity of gastric secretions [37].

**Figure 13:**
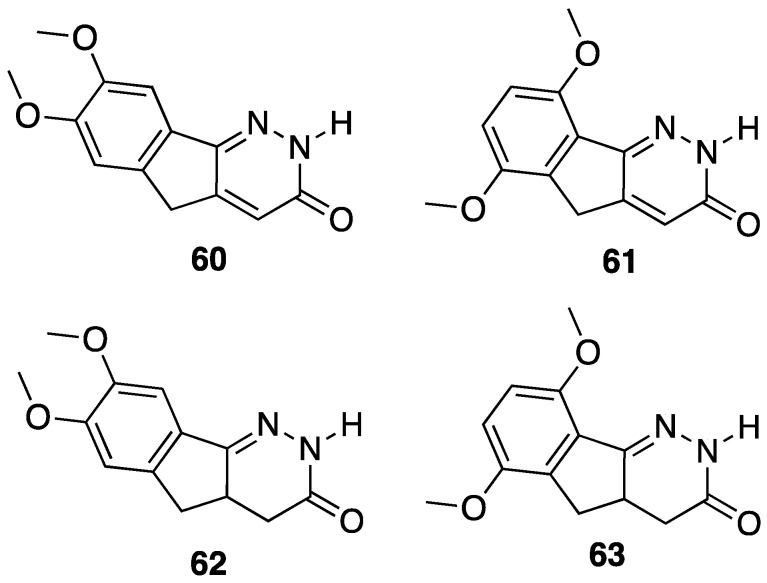
Structure of 5*H*-indeno[1,2-*c*]-3(2*H*)-pyridazinones **60** and **61** and 4,5-dihydro-5*H*-indeno[1,2-*c*]-3(2*H*)-pyridazinones **62** and **63**.

Taking into account the previously described antiulcer properties of tricyclic indenopyridazinone **10** (Figure 4), benzopyrano[4,3-*c*]pyridazin-3(2*H*)ones, and benzo[*h*]cinnolinones **55**–**59**, Barlocco et al., by a scaffold hopping approach, synthesized a series of indenopyridazinones (Figure 13) for antisecretory and antiulcer activity, taking ranitidine as a reference drug [38]. The most interesting compound of this class was the 7,8-dimethoxy-substituted derivative **60**, which, at an oral dose of 30 mg/Kg, still retained significant antisecretory activity (pylorus-ligated rat model). The dihydro-derivatives of **60** and **61**, compounds **62** and **63**, respectively, were more active than (**63**) or comparable (**62**) to the parent compounds. These derivatives were able to prevent hemorrhagic lesions induced by 90% ethanol in rats in a dose-dependent manner. In the indometacine model, they still showed significant activity, albeit not quite as much as in the previous test. An in-depth pharmacological study carried out on compound **60** highlighted that the molecule, contrary to ranitidine, exerted antiulcer activity mainly by improving the integrity of the gastric mucosa while at the same time inhibiting gastric acid hypersecretion induced exclusively by cholinergic pulses. The authors concluded that the mechanism of action for this derivative may involve a neuronal rather than an effectorial mechanism [39].

The antiulcer properties of tricyclic pyridazinones described above are summarized in Table 3.

## 5. Anticancer Activity

According to the WHO, cancer is the second leading cause of death in the world, and was estimated to be responsible for 9.6 million deaths, or 1 in 6 deaths, in 2018. Lung, prostate, colorectal, stomach, and liver cancers are the most common cancers in men, while breast, colorectal, lung, cervical, and thyroid cancers are the most common in women. Cancer is a broad group of diseases characterized by the high proliferation and spread of aberrant cells from their site of origin. Treatment options include surgery, cancer medicines, and/or radiotherapy, which may be given alone or in combination [40]. There are three main periods or “waves” in the history of anticancer drugs, which emerged in succession. Each period is characterized by distinct antitumor activity and toxicity [41]. The first period (classical chemotherapeutics) comprised drugs that primarily interfere with the integrity and/or replication of DNA, such as DNA cross-linking agents [42], mitosis inhibitors or topoisomerase poisons [43,44], and DNA intercalators [45]. In the second period, drugs targeted signaling intermediates that contribute to cancer growth, particularly tyrosine kinase and related inhibitors [46]. The third period encompassed drugs that target a wide range of cellular machineries that are not directly involved in DNA replication or cell division, but are essential for the growth and survival of tumors (proteasome, PARP, protein chaperones, DNA methyltransferase) such as proteasome inhibitors [47], PARP inhibitors [48], chromatin modifiers [49,50], or protein chaperone inhibitors [51]. In this context, several research groups have developed tricyclic pyridazinone-based compounds as anti-proliferative agents, most of which with promising anticancer potential.

**Figure 14:**
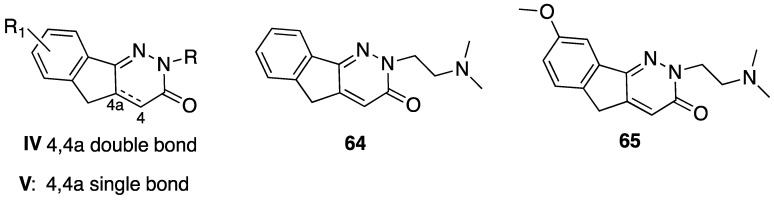
Structure of 5*H*-indeno[1,2-*c*]-3(2*H*)-pyridazinones **64** and **65**.

DNA intercalators are a class of drugs featuring a planar poly-aromatic system that can be inserted between the base pairs of DNA through hydrophobic interactions, van der Waals attractions, hydrogen bonding, and/or charge transfer processes. Such interactions hamper DNA replication. A further structural feature of DNA intercalators is the presence of at least one flexible cationic side chain linked to the heteroaromatic ring, which interacts with the negatively charged phosphate DNA backbone, playing a critical role in the affinity and selectivity of such compounds. On this basis, Cignarella et al. undertook a research program designed to verify whether the tricyclic indenopyridazinone of general formula **IV** and its dihydro analog **V** (Figure 14) could be appropriate scaffolds for the development of DNA-interacting anticancer agents. SAR studies were carried out on 14 compounds by testing their cytotoxic activity on *LoVo* and *LoVo/DX* human colon carcinoma cell lines and on *L1210* and *L1210/CDDP* murine leukemia cell lines, and indicated that the double bond in position 4,4a is essential for activity. Compounds **64** and **65** exhibited comparable activity to cisplatin and melphalan against resistant cell lines and against both sensitive and resistant cell lines, respectively. DNA-binding studies on most of the active compounds have suggested that DNA is an important biological target for the cytotoxic activity of the reported indenopyridazinones. The authors concluded that further investigation is needed to understand the mechanisms (i.e., topoisomerase poisoning and free radical formation) through which **64** and **65** exert their cytotoxic effects [52].

**Figure 15:**
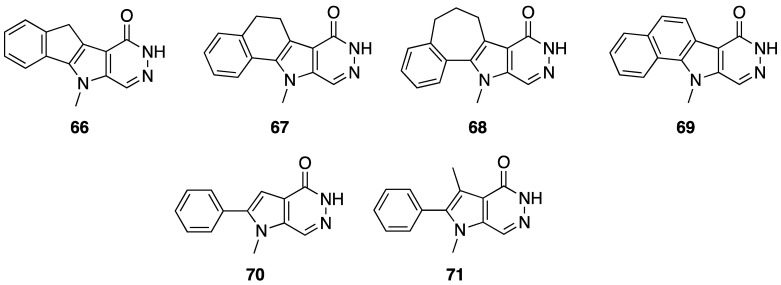
Structure of pyrrolpyridazinones **66**–**71**.

Our interest in the chemistry and pharmacology of polycyclic pyridazinones continued with the design and synthesis of a series of tetracyclic pyrrole[2,3-*d*]pyridazine-4-one derivatives (**66**–**69**, Figure 15) with an almost planar structure, potentially intercalating with DNA. These were evaluated in vitro by the National Cancer Institute against 60 human tumor cell lines derived from nine cancer cell types [53]. Moreover, to compare the variation in biological effects due to the loss of planarity of the ring system, 1-methyl-2-phenylpyrrole[2,3-*d*]pyridazine-4-one **70** and 1,3-dimethyl-2-phenylpyrrole[2,3-*d*]pyridazine-4-one **71**, derived from **66** by the removal or cleavage of the carbon bridge between the phenyl and pyrrole moieties, were synthesized and included in the screening. The biological results showed that, with potency increasing in the order of 71 < 67 ≤ 68 < 69 < 66, the antitumor activities of these compounds were related to the planarity of their ring systems. Among these, the most potent compound **66** showed significant cell line cytotoxicity, particularly against the renal cancer subpanel (growth inhibition: GI_50_ 5.07 μM) and significant potency against leukemia (MOLT-4, SR), non-small-cell lung (NCI-H460), colon (HCT-116), and CNS (SF-295) cancer cells (GI_50_ 3.04–4.32 μM).

**Figure 16:**
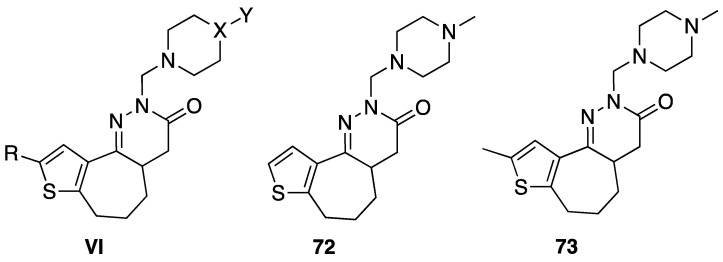
Structure of hexahydrothienocycloheptapyridazinones **72** and **73**.

In a mini-review, Roman encompasses the biological activity of Mannich bases generated from various substrates through the introduction of an aminomethyl function by means of the Mannich reaction. Among various biological properties, Roman extensively reviewed the anticancer and cytotoxic properties of Mannich bases belonging to different chemotypes [54]. A few years earlier, our research group reported a series of twelve tricyclic hexahydrothienocycloheptapyridazinone Mannich bases of general formula **VI** (Figure 16) endowed with anticancer activity. A sulforhodamine B assay (SRB assay) was used for in vitro screening to evaluate growth inhibition properties, at a single 10 μM dose, against sixty different human tumor cell lines. Most of the compounds exhibited no activity on the cell lines tested, but two derivatives, namely **72** and **73**, mildly inhibited the growth of non-small-cell lung cancer (EKVX and HOP-92) and glioblastoma (SNB-75) cell lines, respectively [55].

**Figure 17:**
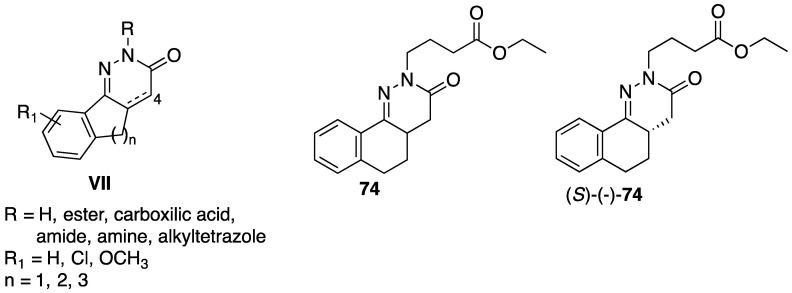
Structure of tetrahydrobenzo[*h*]cinnolinone **74** and (*S*)-(−)-**74**.

Signal transducer and activator of transcription 3 (STAT3) is one of seven members of the STAT family that regulates a variety of biological processes, including proliferation, metastasis, angiogenesis, immune response, and chemoresistance. STAT3 is widely regarded as an attractive target for the development of inhibitors for the treatment of STAT3-related diseases, such as cancers, autoimmune, and inflammatory diseases [56]. Villa et al. reported a series of twenty tricyclic pyridazinones of general formula **VII** (Figure 17) as potential STAT3 inhibitors. All compounds were evaluated in a dual-luciferase assay in human colorectal carcinoma cells, HCT-116, which are characterized by uncontrolled expression of STAT3. After 24 h, the percentages of inhibition were determined at a concentration of 2 μM of the compounds in comparison with cryptotanshinone, a natural compound endowed with apparent similarity to the investigated tricyclic pyridazinones, and endowed with STAT3 inhibition. SAR studies identified the benzocinnolinone derivative **74** as a promising STAT3 inhibitor with 46% inhibitory activity, versus cryptotanshinone endowed with 25% inhibition. Furthermore, enantiomeric separation of compound **74** furnished (*S*)-(−)-**74**, which was twice as potent as (*R*)-(+)-**74** [57].

**Figure 18:**
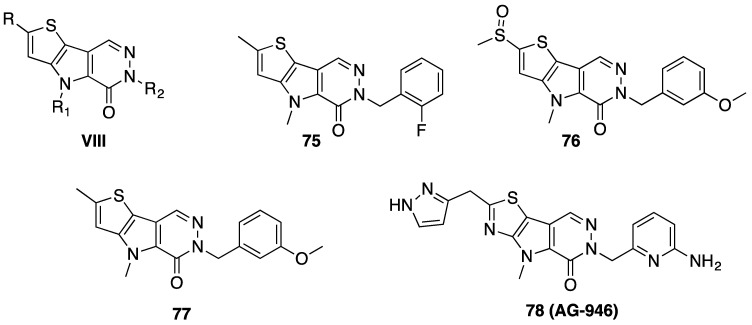
Structure of thieno[3,2-*b*]pyrrole[3,2-*d*]pyridazinones **75**–**77** and **t**hiazolo[5′,4′:4,5]pyrrolo[2,3-*d*]pyridazin-5-one **78** (**AG-946**).

Pyruvate kinase muscle 2 (PKM2), a subtype of pyruvate kinase (PK), regulates the final step of the glycolytic pathway: the transformation of phosphoenolpyruvate and ADP to pyruvate and ATP. It plays a key role in regulating cell metabolism. Many reports highlight the importance of this kinase in cancer progression and different inhibitors, and agonists of PKM2 have been described for future cancer treatment [58]. Activators of PKM2 based on a substituted thieno[3,2-*b*]pyrrole[3,2-*d*]pyridazinone scaffold were described by Thomas et al., from the National Institutes of Health Chemical Genomics Center (general formula **VIII**, Figure 18). These compounds were discovered through a high-throughput quantitative screen of nearly 300.000 small molecules using the firefly luciferase assay system [59]. Compound **75**, with a maximum response at 57 μM of 122% relative to FBP (fructose-1,6-bis-phosphate), showed good and selective potency to activate PKM2 (AC_50_ = 63 nM). Its optimization led to improved compounds in terms of activity and solubility, such as the methylsulfoxide-substituted derivative **76**, which shows activation properties (AC_50_ = 73 nM, 99% maximal response) similar to those of the parent pyridazinone **75** but with improved aqueous solubility (37.4 μg mL^−1^).

Reduced or deficient red-blood-cell-specific forms of PK (PKR) appear to play a crucial role in hemolytic anemias such as sickle cell disease (SCD) or in bone marrow disorders as myelodysplastic syndromes (MDSs). Recently, assuming derivatives **76** and **77** (Figure 18) as reference compounds, Dang et al. described the structure-based drug design of compound **78** (AG-946) as an activator of PK isoforms, including PKR. This strategy allowed the optimization of AG-946 for improved selectivity, especially eliminating the undesirable off-target activity of the PDEIII isoform observed in the original scaffold. The authors summarize the following favorable properties of AG-946: (a) potent activation of human wild-type PK (AC_50_ = 5 nM) and a panel of mutated PK proteins (K410E: AC_50_ = 4 nM) and R510Q (AC_50_ = 7nM); (b) significantly longer half-time of activation (>150-fold) compared with the lead compound **76**; and (c) stabilization of PKR R510Q, an unstable mutant PKR enzyme, and maintenance of its catalytic activity under increasingly denaturing conditions. Currently, AG-946 is being evaluated as a potent, oral, small-molecule allosteric activator of wild-type and mutant PKR in clinical trials for low-risk MDS and SCD [60].

**Figure 19:**
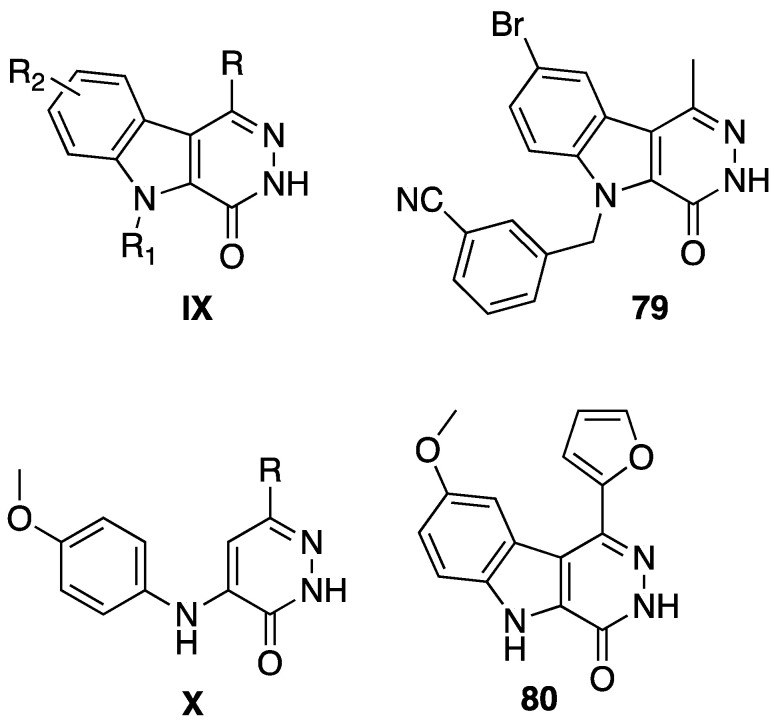
Structure of pyridazino[4,5-*b*]indol-4-ones **79** and **80**.

Bruel et al. reported the SAR study of a series of pyridazino[4,5-*b*]indol-4-ones of general formula **IX** (Figure 19), which were evaluated in selected kinase assays, notably the dual-specificity kinase 1A DYRK1A, the serine/threonine kinase CDK5, and the phosphoinositide 3-kinases PI3Ks [61], described as therapeutic targets in neurodegenerative diseases or cancer [62,63,64]. The authors proposed that, through competitive inhibition at the ATP-binding site, the pyridazino[4,5-*b*]indol-4-one system may be a suitable structure for providing effective interaction with the enzyme. The *N*-5-benzylated derivatives showed promising inhibitory activities against PI3Kα as well as significant anti-proliferative effects in a variety of cell types. Most IC_50_ values were in the μM range. Derivative **79** emerged for its PI3Kα submicromolar affinity (IC_50_ = 0.091 μM). Interestingly, further SAR studies carried out on the pyridazino[4,5-*b*]indol-4-one scaffold, in comparison with the ring-opened analogs of general formula **X**, provided compound **80**, which exhibited submicromolar IC_50_ values against DYRK1A (IC_50_ 0.22 μM) with no activities against the other kinases CDK5/p25, GSK3α/β, and the p110-α isoform of PI3K. Furthermore, derivative **80** displayed anti-proliferative activities in the Huh-7, Caco2, and MDA-MB-231 cell lines [65].

**Figure 20:**
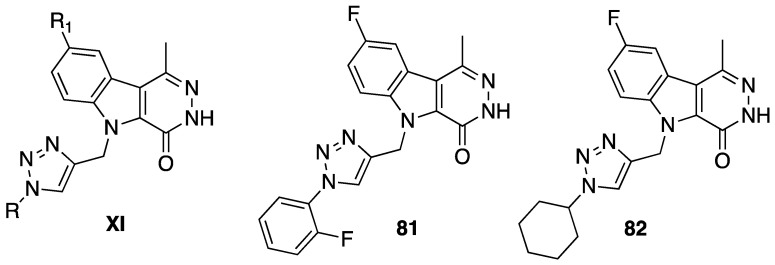
Structure of pyridazino[4,5-*b*]indol-4-one-substituted 1,2,3-triazolylmethyl unit **81** and **82**.

A few years later, Panathur et al. reported a series of thirty compounds of general formula **XI** (Figure 20) featuring the pyridazino[4,5-*b*]indol-4-one scaffold, whose indole nitrogen bore the 1,2,3-triazolylmethyl unit, a motif of several small molecules exhibiting several biological actions, including anticancer activity [66]. Such hybrid derivatives were evaluated in vitro against four cancer cell lines (breast cancer MDA-MB-231 and MCF7, human primary glioblastoma U-87, and human neuroblastoma IMR-32), and most of the compounds showed potent inhibition of cancer cell growth at very low μM concentrations. Compounds **81** and **82**, with IC_50_ values of 0.07 and 0.04 μM, respectively, emerged for their potent activity against the human neuroblastoma cell line IMR-32. Molecular docking studies of all compounds within the ATP-binding site of PI3 kinase (PI3K) suggested that these derivatives could be potential lead inhibitors of the PI3K pathway, which is overexpressed in human tumors.

**Figure 21:**
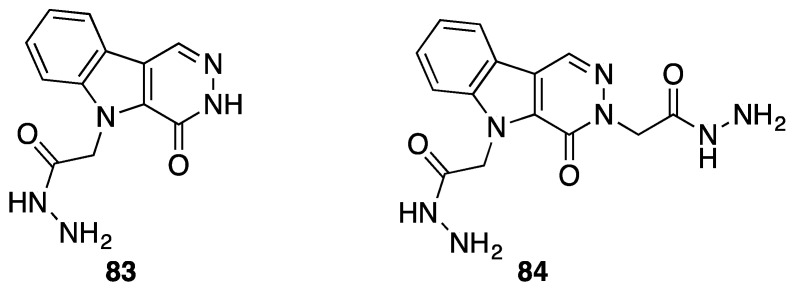
Structure of hydrazide-based pyridazino[4,5-*b*]indol-4-one derivatives **83** and **84**.

Recently, Sarhan et al. described further pyridazino[4,5-*b*]indol-4-ones as new PI3K inhibitors for breast cancer therapy [67]. The hydrazides **83** and **84** (Figure 21) had promising, potent, and significant cytotoxic activity against the MCF-7 breast cancer cell line, with IC_50_ values of 4.25 and 5.35 μM, respectively, compared to the standard drug 5-FU (IC_50_ 6.98 μM).

**Figure 22:**
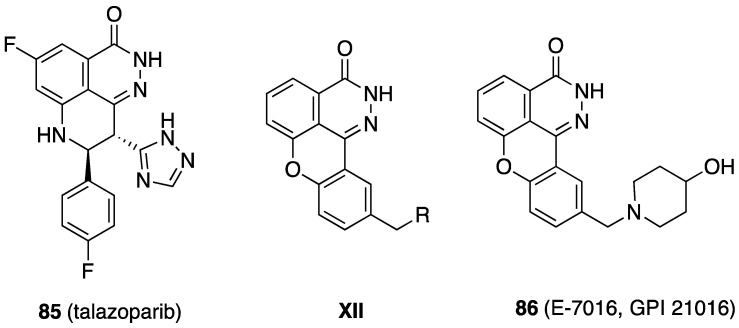
Structure of talazoparib (**85**) and E-7016 (**86**).

In the last decade, poly(ADP-ribose) polymerases (PARPs) have emerged as a new target in cancer treatment [68]. PARPs are known to catalyze the process of PARylation, transferring ADP-ribose residues from nicotinamide adenine dinucleotide (NAD) to target substrates. PARP-1, a subtype of a family of at least seventeen members, is involved in the maintenance of genomic stability, DNA repair, and regulating DNA transcriptional processes. Several PARP-targeted agents, including niraparib, talazoparib, pamiparib, and fluzoparib, are currently approved for the treatment of malignant tumors, such as pancreatic cancer, breast cancer, and prostate cancer [69]. From a structural point of view, several PARP-1 inhibitors incorporate a bicyclic pyridazinone core or even a tricyclic core, such as talazoparib **85** (Figure 22), which was developed by Pfizer and approved to treat adults with deleterious or suspected germline *BRCA*-mutated, HER2-negative, locally advanced, or metastatic breast cancer [14,70]. Guilford/MGI/Eisai Pharmaceuticals discovered a series of tetracyclic PARP-1 inhibitors of general formula **XII** (Figure 22), featuring the pyridazinone core assembled with a xanthene unit. Currently, compound **86**, namely E-7016 (PARP-1 IC_50_ = 0.04 μM), is under evaluation in combination with temozolomide in patients with advanced solid tumors or with wild-type BRAF stage IV or unresectable stage III melanoma [71].

**Figure 23:**
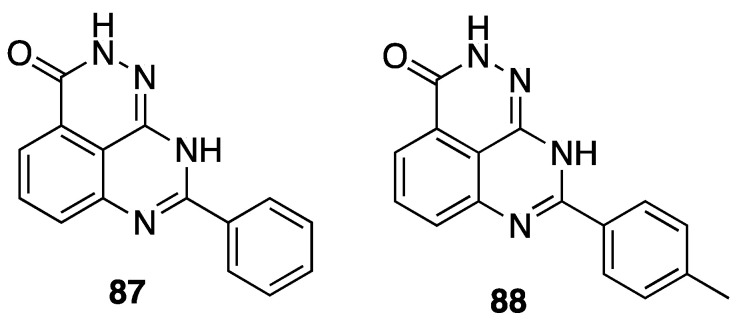
Structure of pyridazino[3,4,5-de]quinazolin-3(2*H*)-ones **87** and **88**.

Considering the pharmacological relevance of pyridazinones along with quinazolines as PARP-1 inhibitors, Li et al. designed and synthesized a series of eight hybrid pyridazino[3,4,5-de]quinazolin-3(2*H*)-ones. All derivatives were tested as PARP-1 inhibitors, and analogs **87** and **88** (Figure 23), endowed with IC_50_ = 0.148 μM and 0.152 μM, respectively, were reported as the most active compounds of the series with neuroprotective effects in a PC12 cell model injured by H_2_O_2_ [72].

Dihydrofolate reductase (DHFR) is a ubiquitous enzyme that catalyzes the reduction of folic acid to tetrahydro-folic acid (THF) using NADPH as a cofactor. DHFR pairs with thymidylate synthase (TS), which catalyzes the reductive methylation of deoxyuridine monophosphate (dUMP) in deoxythymidine monophosphate (dTMP) using *N*^5^-*N*^10^-methylenetetrahydrofolate (5,10-methylene THF) as a cofactor. Inhibition of DHFR, which is responsible for the generation of raw materials for DNA replication, is the basis for the treatment of several infectious diseases and is the target of antibacterial drugs like trimethoprim as well as the anticancer drug methotrexate [73].

**Figure 24:**
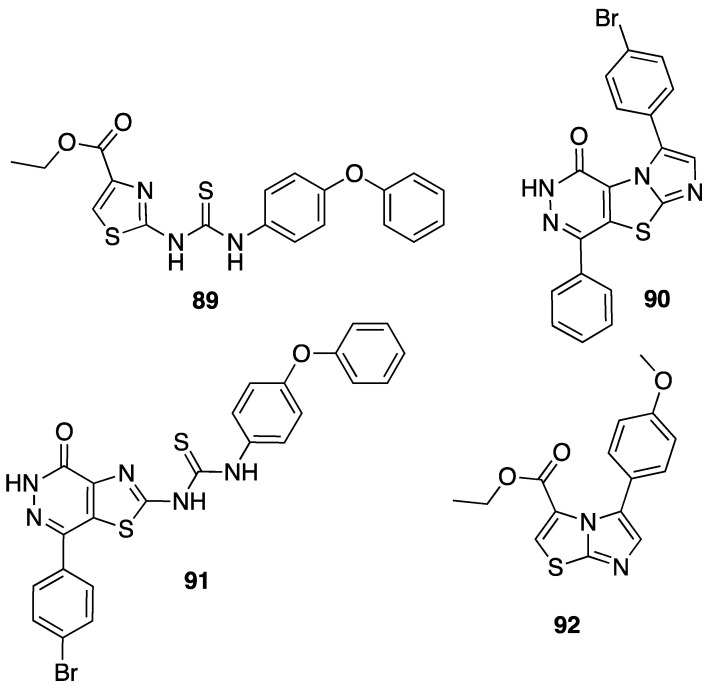
Structure of 1,3-thiazole-based compounds **89** and **92** and polycyclic pyridazinones **90** and **91**.

Using lead compound **89** (Figure 24), which carries carboxyl ester function along with a thioureido function and has an IC_50_ inhibition affinity of 0.05 μM, as a reference, Ewida et al. designed and synthesized a series of 1,3-thiazole-containing heterocycles acting at the DHFR enzyme active site. Modification carried out on compound **89**, notably the replacement of the thiazole scaffold with imidazo[2,1-*b*]thiazole and the annexation of carboxyl ester function into a pyridazinone unit, provided the tricyclic imidazo[2′,1′:2,3]thiazolo[4,5-*d*]pyridazinone **90** with an IC_50_ value of 0.06 μM [74]. Interestingly, SAR studies have shown that ring annexation of the active 1,3-thiazole ring of **89** in the bicyclic thiazolo[4,5-*d*]pyridazinone (i.e., **91**, IC_50_ = 0.35 μM) or imidazo[2,1-*b*]thiazoles (i.e., **92**, IC_50_ = 49.67 μM) reduced DHFR inhibition activity, whereas the formation of the tricyclic imidazo[2′,1′:2,3]thiazolo[4,5-*d*]pyridazinone increased the potency, with **90** being the most representative of these novel DHFR inhibitors. Compound **90** proved to be lethal for OVCAR-3 ovarian cancer and MDA-MB-435 melanoma cells at IC_50_ = 0.32 and 0.46 μM, respectively.

The anticancer properties of the representative tricyclic pyridazinones described above are summarized in Table 4.

## 6. Antidiabetic Activity

**Figure 25:**
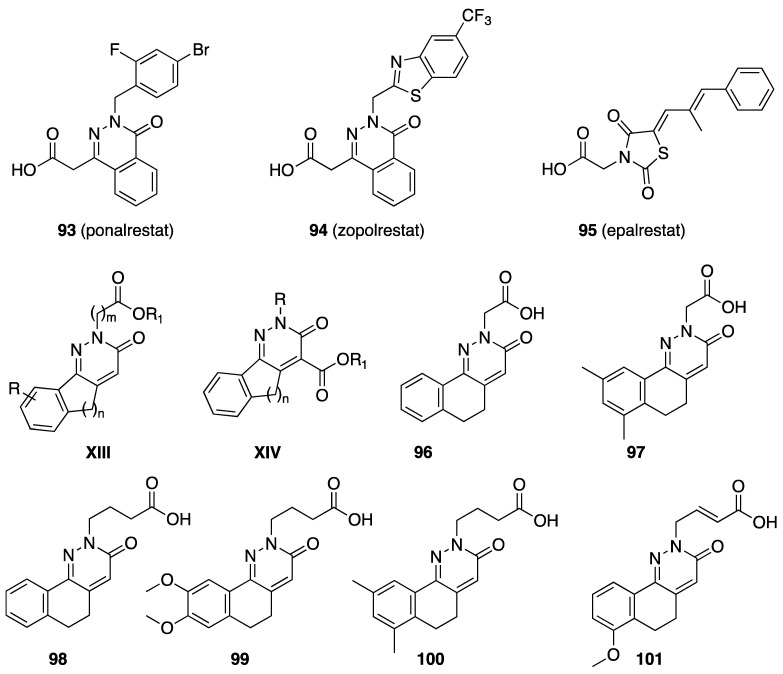
Structure of ponalrestat (**93**), zopolrestat (**94**), epalrestat (**95**), and benzocinnolinones **96**–**101**.

Diabetes is a group of metabolic disorders affecting more than 400 million people worldwide, becoming a global public health concern [75]. The hyperglycemic condition, in both type 1 and 2 diabetes, is associated with long-term complications and dysfunction of vital organs such as the kidney, heart, nerves, and eyes [76]. There is growing evidence that the toxic effects of hyperglycemia are associated with the activation of the polyol metabolic pathway, where glucose is converted to sorbitol by the first enzyme of the polyol pathway, aldose reductase (ALR2), with the oxidation of NADPH. Sorbitol is subsequently converted to fructose by the enzyme sorbitol dehydrogenase (SDH), with concomitant reduction of NAD^+^ [77]. ALR2 has been considered for many years as a valid target for the development of drugs capable of preventing or delaying the appearance of long-term diabetic complications without the risk of hypoglycemia. This is because ALR2 inhibitors have no effects on plasma glucose [78,79]. Different ALR2 inhibitors have been studied as potential drug candidates, such as the pyridazinone derivatives ponalrestat (**93**) and zopolrestat (**94**) for the treatment of diabetic complications, with IC_50_ values against ALR2 of 20 and 2.1 nM, respectively (Figure 25) [80]. However, there are several problems associated with ALR2 inhibition therapy, including poor selectivity and loss of efficacy with prolonged treatment. To date, epalrestat (**95**) is the only compound successfully marketed in Japan, India, and China to treat diabetic neuropathy [78].

Within this frame, Barlocco et al. described the SAR studies of a series of tricyclic pyridazinones bearing acidic side chains at the N_2_ or C_4_ position, of general formulas **XIII** and **XIV** (Figure 25), which were evaluated as ALR2 inhibitors; sorbitol dehydrogenase and other enzymes are not implicated in the polyol pathway like aldehyde reductase and glutathione reductase. From this study, the acetic and butyric acid benzocinnolinones **96**–**101** emerged as the most active and selective ALR2 inhibitors, with IC_50_ values ranging from 4.25 (**101**) to 12.6 μM (**96**), even if less effective than ponalrestat or zopolrestat which act in the nM range [81,82].

**Figure 26:**
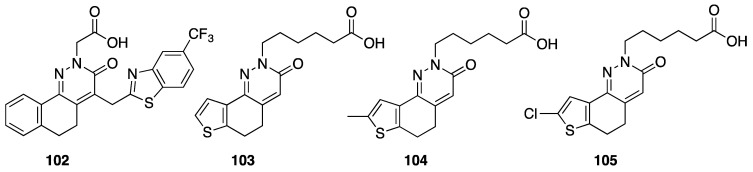
Structure of benzocinnolinone **102** and thienocinnolinones **103**–**105**.

Using a structure-based drug design strategy and the ALR2 inhibitor zopolrestat (**94**) as a reference compound, along with the benzocinnolinone derivative **96**, Rastelli et al. discovered the hybrid compound **102** (Figure 26), which is two orders of magnitude more potent than the model **96** (IC_50_ = 0.15 μM) [83]. Within this frame, some years later, our group disclosed a series of thienocinnolinone derivatives with activity as ALR2 inhibitors. Compounds **103**–**105** (Figure 26), featuring a thienocinnolinone core linked through a pentamethylene spacer to a carboxylic function, exhibited IC_50_ values of 7.6, 18, and 31.4 μM, respectively [84].

**Figure 27:**
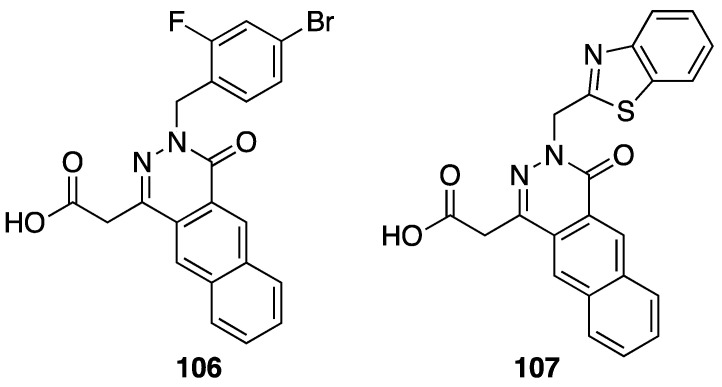
Structure of tricyclic pyridazinones **106** and **107**.

Further, tricyclic pyridazinones structurally related to ponalrestat and zopolrestat were disclosed by Pfizer [85]. Compounds **106** and **107** (Figure 27) exhibited ALR2 inhibition activities of 420 and 46 nM, respectively.

ALR2 enzyme inhibition data for tricyclic pyridazinones described above are summarized in Table 5.

## 7. Anxiety Disorders

In the *Diagnostic and Statistical Manual of Mental Disorders* of the American Psychiatric Association (DSM-5), anxiety disorders include generalized anxiety disorder, separation anxiety disorder, social phobia, specific phobia, panic disorder, agoraphobia, and selective mutism. Benzodiazepines are efficacious drugs for treating anxiety disorders, and several of them have received approval for clinical use. The pharmacological effects of benzodiazepines result from their affinity for a specific binding site on the GABA_A_ receptor, the benzodiazepine site (BZR). They exhibit a continuum of intrinsic activity, ranging from full agonists (positive allosteric modulators of GABA-evoked Cl^−^ currents with anxiolytic, hypnotic, and anticonvulsant activities), antagonists with no intrinsic activity, and inverse agonists (negative allosteric modulators of GABA-evoked Cl^−^ currents with proconvulsant, convulsant, and anxiogenic activities) [86].

**Figure 28:**
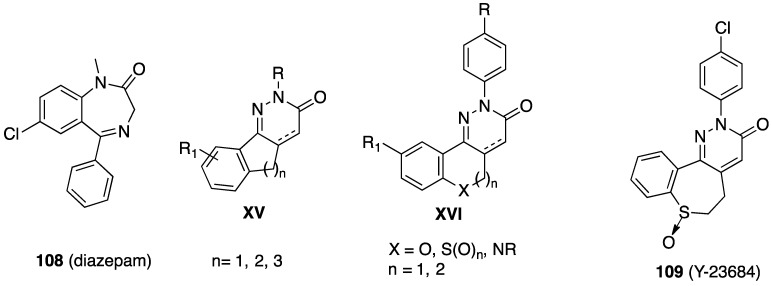
Structure of diazepam (**108**) and benzothiepino[5,4-*c*]pyridazine-3(2*H*)-one-7-oxide **109**.

Most of the drugs in clinical use, such as diazepam (**108**, Figure 28), are full agonists at the BZR and have anticonvulsant, sedative, and muscle-relaxant effects along with anxiolytic properties [14]. Partial agonists of the BZR such as imidazenil offer some theoretical and practical advantages over full agonists, including fewer side effects such as sedation, ataxia, and abuse potential, even though no GABA_A_ partial agonist has received approval for treating anxiety disorders. In the past, several research groups, both from academia and industry, dedicated many efforts to discovering drugs targeting the BZR, and a few structural classes of nonbenzodiazepine compounds showed desired anxiolytic or other pharmacological activities in vivo, such as zoplicone and zolpidem, which were marketed to treat insomnia [14].

As part of this research, Yoshitomi Pharmaceutical Industrie has been involved for several years in the design and synthesis of BZR ligands of the general formulas **XV** and **XVI**, incorporating a tricyclic pyridazinone system (Figure 28). SAR studies of synthesized compounds identified the benzothiepino[5,4-*c*]pyridazine-3(2*H*)-one-7-oxide **109** (Y-23684) as a partial BZR agonist, exhibiting K_i_ = 42 nM versus diazepam, K_i_ = 5.8 nM (radioligand-binding assay with [^3^H]diazepam in the cerebral cortex of rats). An in-depth pharmacological study conducted on this compound in comparison with diazepam suggested that **109** would be a potent and selective anxiolytic agent in humans with fewer side effects than conventional BZ-anxiolytics [87].

**Figure 29:**
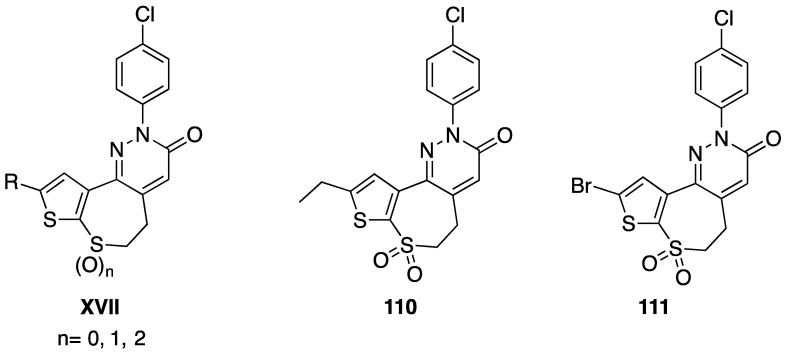
Structure of thieno[2′,3′:2,3]thiepino[4,5-*c*]pyridazine-3(2*H*)-ones **110** and **111**.

Taking into consideration the pharmacological relevance of these compounds, further tricyclic pyridazinones have been designed and synthesized, taking **109** as a reference compound. Isosteric replacement of the condensed benzene ring of **109** with a thiophene ring, along with the introduction of various substituents into the 9-position of the condensed-ring system, and variation of the oxidation level of the sulfoxide function (from sulfoxide to sulfide or sulfone) at the 7-position of the same system, gave several thieno[2′,3′:2,3]thiepino[4,5-*c*]pyridazine-3(2*H*)-ones of general formula **XVII** (Figure 29) as potent ligands at the BZR, most of them endowed with K_i_ values ranging from 0.61 to 6.8 nM. Modifications carried out on the lead compound **109** had a remarkable impact both on affinity values to the BZR and in vivo tests. Electrophysiological studies conducted in vitro (GABA-induced chloride current in frog sensory neurons) along with in vivo tests such as an anticonvulsant test (anti-BCL test), anticonflict test (water-lick test), and rotarod test, indicated that the scaffold thieno[2′,3′:2,3]thiepino[4,5-*c*]pyridazine-3(2*H*)-one provided compounds with a broad spectrum of activities ranging from full agonists to partial agonists. Compounds **110** (K_i_ = 1.1 nM) and **111** (K_i_ = 0.61 nM), featuring a sulfone function, exhibited significantly greater BZR affinity than diazepam and were classified as a full agonist and partial agonist, respectively [87].

**Figure 30:**
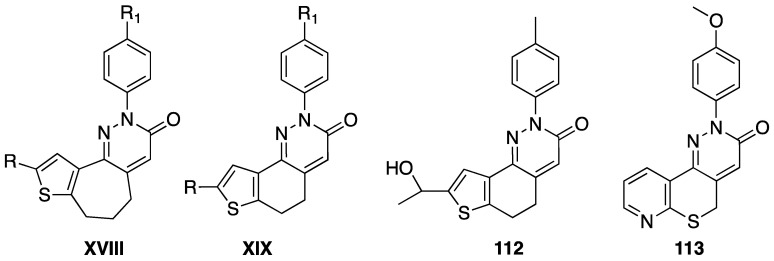
Structure of thienocinnolinone **112** and pyrido-thiopyrano-pyridazinone **113**.

The SAR studies carried out on such tricyclic pyridazinones were extended to thieno[2′,3′:6,7]cyclohepta[1,2-*c*]pyridazine-3-(2*H*)ones and thieno[2,3-*h*]cinnoline-3(2*H*)ones of general formulas **XVIII** and **XIX**, respectively (Figure 30) [88]. Among the tested compounds, several had affinity for the BZR in the nM range. Particularly, the partial agonist **112** exhibited an anxioselective feature.

In this context, some years later, Primofiore et al. designed, synthesized, and evaluated the binding affinity of a series of pyrido[3′,2′:5,6]thiopyrano[4,3-*c*]pyridazine-3(2*H*,5*H*)-one [89] to the BZR. The target compounds exhibited an affinity in the micromolar/submicromolar range, with derivative **113** (Figure 30) exhibiting the most potent affinity (K_i_ = 0.695 μM).

The BZR-binding affinities of the tricyclic pyridazinones described above are summarized in Table 6.

## 8. Antiviral Activity

The integrase (IN) enzyme represents an important chemotherapeutic target as its inhibition blocks infection by human immunodeficiency virus type 1 (HIV-1), which is responsible for acquired immune deficiency syndrome (AIDS). In fact, integrase strand-transfer inhibitors (INSTIs) represent one of the most significant advances in HIV care, being the latest combination antiretroviral therapy (cART) drugs developed [90]. Integrase is one of three core enzymes encoded by the HIV genome and is essential for the integration of viral genetic material into the host cell DNA and successful replication of the virus. INSTIs bind to the integrase–viral DNA complex in the catalytic core domain. As a result, they interfere with the integration of viral DNA into host DNA. It has been proposed that INSTIs bind to key metals that are involved in the action of integrase and thus block the viral enzyme [91]. Raltegravir and, more recently, elvitegravir, dolutegravir, and cabotegravir were approved compounds within cART formulations [14,90].

**Figure 31:**
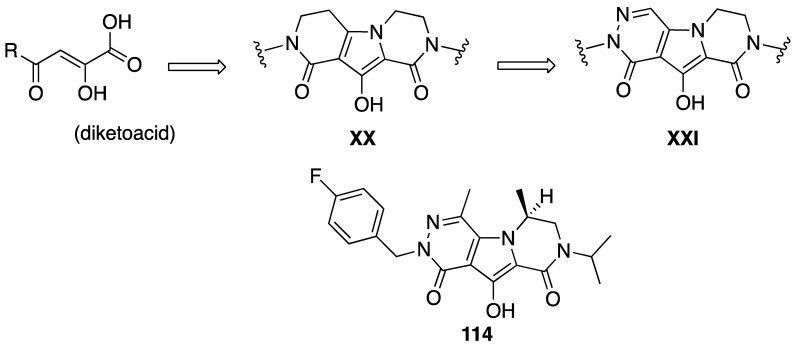
Structure of 10-hydroxy-7,8-dihydropyrazino[1′,2′:1,5]pyrrolo[2,3-*d*]pyridazine-1,9(2*H*,6*H*)-dione **114**.

Wiscount et al., from Merck laboratories, described a tricyclic hydroxypyrrole scaffold **XX** (Figure 31) as a new mimetic of the metal-binding pharmacophore of diketoacid integrase inhibitors. In spite of the unique opportunities of such compounds to maintain activity against integrase mutants, in pharmacokinetic experiments, they were susceptible to air oxidation. The authors assumed that the tricyclic pyridazinone scaffold **XXI** could provide compounds with improved chemical stability and better pharmacokinetic properties, keeping the same activity against the integrase enzyme. Among the synthesized compounds, analog **114** inhibited integrase strand-transfer activity with an IC_50_ less than 10 nM and also inhibited HIV-1 cell culture replication with an IC_95_ value of 35 nM in the presence of 50% normal human serum. This compound may represent a promising lead for further optimization studies toward second-generation integrase strand-transfer inhibitors, having shown modest pharmacokinetic properties in rats (*iv t*_1/2_ = 5.3 h, *F* = 17%) [92]. No further data are available for this class of tricyclic pyridazinones.

## 9. Other Biological Activities

### 9.1. Matrix Metalloproteinase Inhibitors

**Figure 32:**
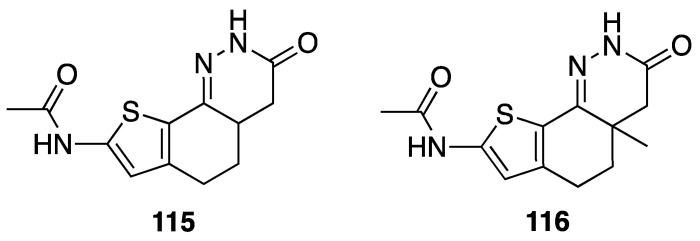
Structure of thieno[3,2-*h*]cinnolinones **115** and **116**.

Matrix metalloproteinases (MMPs) belong to a family of zinc^2+^-dependent enzymes including about 28 members involved in the degradation of different protein targets. MMPs play a major role in the pathogenesis of cancer, autoimmune diseases, cardiovascular diseases, inflammation such as periodontal diseases, and neurodegenerative disease states [93,94]. Our group reported a series of thieno[3,2-*h*]cinnolinones which were tested against the Met80-Gly242 domain of human neutrophil collagenase (MMP-8) expressed in *Escherichia coli*. Compounds **115** and **116** (Figure 32), featuring an NH-acetyl group in position 8 of the thieno[3,2-*h*]cinnolinone scaffold, were found to be the most potent analogs with IC_50_ values of 17 and 36 μM, respectively [95].

### 9.2. Phosphodiesterase 5 Inhibitors

**Figure 33:**
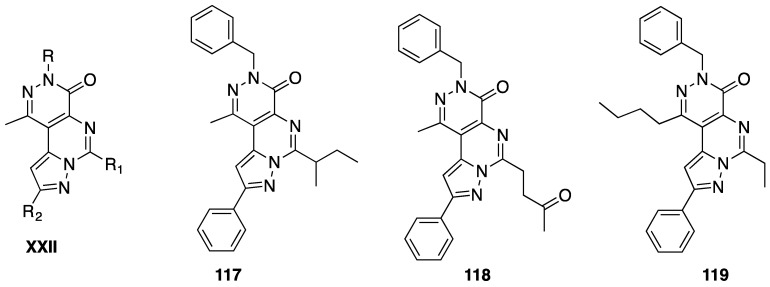
Structure of pyrazolopyrimidopyridazinones **117**–**119**.

Phosphodiesterase 5 (PDE5) is 1 of the 11 members of the cyclic nucleotide phosphodiesterase (PDE) family. It specifically targets cyclic guanosine monophosphate (cGMP), produced by nitric oxide-driven activation of the soluble guanylyl cyclase, an important mediator of vasodilation. PDE5 inhibition extends the duration of cGMP and promotes vasodilation. PDE5 inhibitors, including sildenafil and tadalafil, are widely used to treat erectile dysfunction, pulmonary arterial hypertension, and certain urological disorders. Preclinical studies have also revealed promising effects of PDE5 inhibitors in the treatment of myocardial infarction, cardiac hypertrophy, heart failure, cancer and anticancer-drug-associated cardiotoxicity, Alzheimer’s disease, and other aging-related conditions [96]. The research group of Dal Piaz and Giovannoni synthesized a series of tricyclic pyrazolopyrimidopyridazinones of general formula **XXII** (Figure 33) with potent and selective PDE5 inhibition properties as potential agents for the treatment of erectile dysfunction. Such compounds were evaluated as inhibitors for PDE5 along with PDE6, the off-target isoform of sildenafil. Several of the tested compounds showed IC_50_ values in the low nM range compared to sildenafil (PDE5 IC_50_ = 0.020 μM, PDE6 IC_50_ = 0.040 μM) and high selectivity versus the PDE6 isoform. Compounds **117** and **118** emerged as the most interesting compounds (**117**: PDE5 IC_50_ = 0.034 μM, PDE6 IC_50_ = 42.5 μM; **118**: PDE5 IC_50_ = 0.0083 μM, PDE6 IC_50_ = 22.8 μM) [97,98].

### 9.3. Adenosine Inhibitors

Adenosine is a ubiquitous endogenous autacoid. It is involved in a number of physiological and pathological effects in the cardiovascular, renal, nervous, and immune systems, through G-protein-coupled receptors, namely A_1_, A_2A_ A_2B_, and A_3_ adenosine receptors. Several types of agonists, partial agonists or antagonists, and allosteric substances have been identified for these receptors as new therapeutic drug candidates [99]. Giovannoni et al. synthesized a new series of tricyclic pyrazolopyrimidopyridazinones **XXII** (Figure 33) and evaluated their affinity towards human A_1_, A_2A_, and A_3_ adenosine receptors [100,101]. Most of the tested compounds showed high selectivity of action towards the A_1_ receptor subtype and high affinity with K_i_ values in the low nM range. The most interesting compound was **119** (hA_1_ K_i_ = 1.4 nM, selectivity 7%, and 20% of inhibition at 10 μM for hA_2A_ and hA_3_, respectively). Further pharmacological studies carried out on **119** evidenced antagonistic properties at the A_1_ receptor and antiamnesic effects in the mouse passive avoidance test at doses of 10 and 30 mg/Kg.

### 9.4. Translocator Protein 18 kDa (TSPO) Ligands

**Figure 34:**
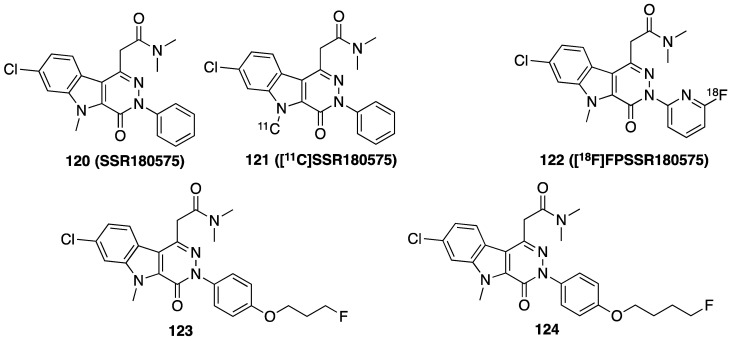
Structure of pyridazino[4,5-*b*]indole-1-acetamides **120**–**124**.

An interesting biological target for the discovery of promising neuropsychotropic compounds is the translocator protein 18 kDa (TSPO), formerly known as the peripheral benzodiazepine receptor (PBR). This protein is predominantly localized in the mitochondrial membrane and is found in both the peripheral and central nervous systems. It is involved in cholesterol translocation, which is essential for the production of steroid hormones and neurosteroids [102]. TSPO is overexpressed in neuroinflammation conditions. It may be a suitable neuroinflammation biomarker and a target for positron emission tomography (PET) and single-photon emission computed tomography (SPECT) imaging. TSPO also has a role in apoptosis, cell proliferation and differentiation and many other physiological functions such as porphyrin transport and heme biosynthesis [103]. Numerous classes of selective synthetic ligands have been reported to further the understanding of TSPO functions in neurodegenerative and neuroinflammatory conditions [104]. Compound **120** (SSR180575), belonging to the chemical class of pyridazino[4,5-*b*]indole-1-acetamides (Figure 34), binds to the TSPO with a high affinity (K_i_ = 0.83 nM). It represents an attractive lead for the development of TSPO ligands as therapeutic drugs. This compound has also been tagged with carbon-11 (**121**) and has shown encouraging in vivo PET imaging properties in both rodents and non-human primates. It also has the ability to support neuronal survival and regeneration in animal models of axotomy and neuropathy by facilitating the local synthesis of neurosteroids [103]. A series of related compounds exhibiting strong affinity, with K_i_ values similar to those of SSR180575 (**120**), were discovered by functionalization at the *N*-3 position, allowing for substitution with F-18. Among them, derivative [^18^F]FPSSR180575 (**122**) emerged as a promising radioligand for in vivo PET imaging. Further SAR studies carried out on SSR180575 (**120**) provided several derivatives exhibiting potent TSPO affinity, with K_i_ values in the nM and sub-nM ranges. Among such derivatives, compounds **123** and **124** (**123**: K_i_ = 0.40 nM; **124**: K_i_ = 0.42 nM) emerged as promising candidates for drug development. They also offer unique opportunities for fluorine-18 labeling and in vivo PET imaging of neuroinflammation [104].

### 9.5. Multitarget-Directed Ligands

Alzheimer’s disease (AD) is a chronic, progressive, and incurable neurological disorder that affects millions of people worldwide. Notably, low levels of acetylcholine in the cerebral cortex and other brain areas appear to play a critical role in the development of cognitive and neurodegenerative disorders in AD (cholinergic hypothesis). Acetylcholinesterase inhibitors (AChEIs), such as donepezil, rivastigmine, or galantamine, or *N*-methyl-D-aspartate receptor antagonists, such as memantine, are currently the pillar of pharmacological therapy for AD [14].

Taking into consideration the knowledge of the multifactorial nature of AD, along with the inability of current therapies to stop the progression of the disease, several efforts have been made to identify novel and safer AChEIs or new biological targets to treat AD. Within this framework, the multitarget-directed ligand (MTDL) strategy “one molecule, multiple targets” has been applied to the design and synthesis of new drug candidates capable of interacting with multiple targets involved in the pathogenesis of AD [105].

**Figure 35:**
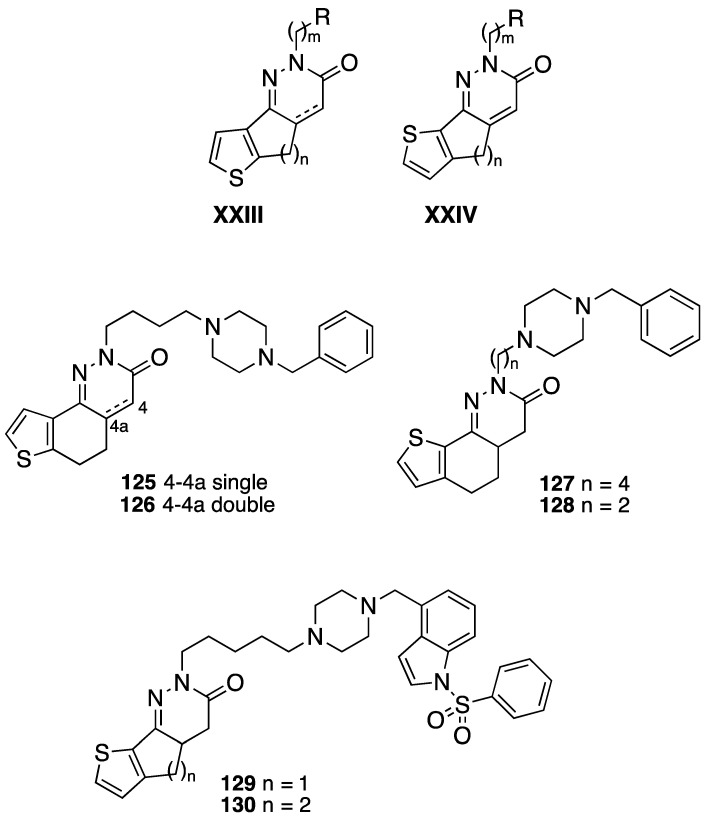
Structure of thieno-cycloalkyl-pyridazinones **125**–**130**.

Our research group focused on the design, synthesis, and pharmacological evaluation of a library of thienocycloalkylpyridazinones of general formulas **XXIII** and **XXIV** (Figure 35) using the MTDL approach, in the pursuit of potential AD treatment discovery [106]. These compounds featured pyridazinone-based tricyclic scaffolds connected through alkyl chains of variable length to proper amine moieties for G-protein-coupled receptors (especially serotonin receptors), and cholinesterase (AChE and BChE) molecular recognition. Notably, serotonin receptors, especially 5-HT_1A_, 5-HT_4_, 5-HT_6_, and 5-HT_7_, have attracted much interest as important players in influencing different aspects of cognitive deficits, learning and memory decline in AD. Our SAR studies carried out on the tricyclic pyridazinones **XXIII** and **XXIV** allowed us to identify several compounds showing AChE/BChE inhibition with a wide range of potencies, depending on the correct combination of the linker and the amine moiety. Compounds **125** and **126**, containing the thieno[2,3-*h*]cinnolinone nucleus, exhibited high affinity for AChE with low affinity for BChE (**125**: AChE IC_50_ = 0.86 μM, BChE IC_50_ = 3.3 μM; **126**: AChE IC_50_ = 0.64 μM, BChE IC_50_ = 2.1 μM). Interestingly, compound **127**, which contains the thieno[3,2-*h*]cinnolinone core, maintained an AChE IC_50_ value very similar to that of its positional isomer **125**, exhibiting improved selectivity versus BChE (AChE IC_50_ = 0.75 μM, BChE 45% of inhibition at 10 μM). In general, our SAR study revealed an objective difficulty in obtaining ligands with potent and mixed properties at cholinesterase enzyme and serotonin receptors, i.e., compounds **127** and **128**, exhibiting the highest potency at AChE (**128**: AChE IC_50_ = 0.17 μM, BChE IC_50_ = 5.1 μM) among all tested compounds, had poor or not detectable affinity for serotonin 5-HT_1A_, 5-HT_4_, 5-HT_6_ and 5-HT_7_ receptors. Replacement of the benzyl linked to the piperazine with the phenylsulfonyl indole moiety significantly improved the affinity for 5HT_6_ and selectivity over other serotoninergic receptors, with a slight reduction in inhibitory activity at AChE. Compound **130**, bearing a phenylsulfonyl-indol-piperazine motif with a thieno[3,2-*h*]cinnolinone core connected by a pentamethylene linker, was identified as the best compromise with respect to multitarget affinity (AChE IC_50_ = 4.44 μM, 5-HT_6_ Ki = 63 nM). On the other hand, the replacement of the thieno[3,2-*h*]cinnolinone nucleus in **130** with 2,4,4a,5-tetrahydro-3*H*-thieno[3′,2′:4,5]cyclopenta[1,2-*c*]pyridazin-3-one resulted in compound **129**, the most potent and selective ligand for the 5-HT_6_ receptor subtype, reaching an outstanding K_i_ value in the low nM range (AChE IC_50_ = 6.9 μM, 5-HT_6_ Ki = 8.4 nM).

### 9.6. PKM2 Activators to Reduce Photoreceptor Apoptosis

**Figure 36:**
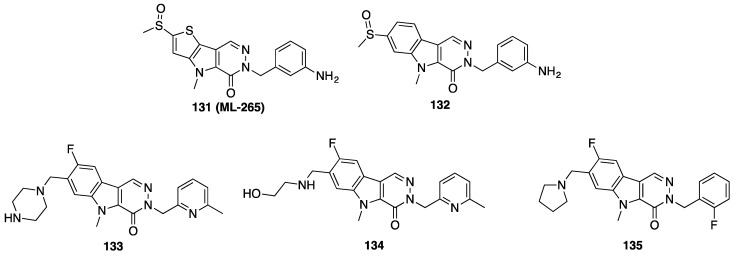
Structure of thieno[3,2-*b*]pyrrole[3,2-*d*]pyridazinone **131** and pyridazino[4,5-*b*]indol-4-one **132**–**135**.

Photoreceptor cell degeneration in many retinal disorders causes significant diminution of the retina’s ability to detect light, resulting in loss of vision. In order to restore vision, ocular regenerative medicine uses neuroprotective therapy, gene therapy, cell replacement therapy, and visual prostheses. Several of these treatments are still at an early stage. They require extensive additional preclinical and clinical studies, before restoration of visual function can be observed [107]. Besirli’s research group reported that the anticancer thieno[3,2-*b*]pyrrole[3,2-*d*]pyridazinone **131** (ML-265) (Figure 36), a potent activator of PKM2 (AC_50_ 70 nM, maximum activation of 292%), exhibited photoreceptor neuroprotection, evaluated in vivo in the rat retina, reducing entry into apoptosis and improving photoreceptor viability in both in vitro and in vivo models of outer retinal stress. In spite of such promising results demonstrating the beneficial use of PKM2 activators as neuroprotective agents, ML-265 suffers from a lack of aqueous solubility and the presence of structural alerts (methyl sulfoxide and aniline functional groups) that prevent it from being further developed as an ideal intraocular clinical candidate. SAR studies carried out on compound ML-265 identified pyridazino[4,5-*b*]indol-4-one **132** as a potent activator of PKM2 with AC_50_ and maximal activation values close to the parent compound ML-265. Compound **132** prevented photoreceptor apoptosis in commonly used in vitro and in vivo preclinical models of outer retinal stress. Such results paved the way for novel pyridazino[4,5-*b*]indol-4-one PKM2 activators, whose best representatives are compounds **133**–**135** (Figure 36), retaining nM potency versus PKM2 (62–64 nM range), with 2- to 7-fold greater aqueous solubilities than ML265. The authors highlighted the importance of their work in providing such novel PKM2 activators as the basis for new therapeutic agents useful for treating retinal degenerative diseases [108].

The different biological properties of the representative tricyclic pyridazinones described above are summarized in Table 7.

## 10. Conclusions

Heterocyclic compounds, especially those containing one or more nitrogen atoms in their structures, have always attracted attention for their therapeutic importance. Among them, the six-membered nitrogen-containing heterocycle, pyridazin-3(2*H*)one, represents an interesting scaffold in several drugs, both those bearing various substituents and forming linear or, more often, angular polycyclic systems, when condensed with other (hetero)cyclic systems.

In particular, this review focused on pyridazin-3(2*H*)one-based tricyclic or tetracyclic molecules and provides an overview of their main potential therapeutic applications, although these types of templates have been tested for many other biological targets. In addition, to highlight the therapeutic potential of these compounds, SAR studies using both in vitro and in vivo assays have been reported.

The pharmacological relevance of tricyclic- or tetracyclic-based pyridazin-3(2*H*)one molecules has been well documented since the last century. However, the identification of new polycyclic pyridazin-3(2*H*)one-based molecules with these structures is still an important field of research due to their pharmacological ductility, responsible for their anti-inflammatory, anticancer, antimicrobial, and many other pharmacological activities. Therefore, the drug design and discovery process of novel polycyclic pyridazin-3(2*H*)one-based scaffolds could still prove useful in the future to identify new molecules able to modulate known and new biological targets involved in different diseases.

In conclusion, pyridazin-3(2*H*)one-based scaffolds have proven to be versatile and valuable in medicinal chemistry. Their sometimes-easy synthesis and broad spectrum of biological activities have made them attractive candidates for drug development across a range of therapeutic areas. While significant progress has been made in the understanding of the SARs of various pyridazinone derivatives, further research is essential to unlock the full therapeutic potential of these compounds. Future efforts should focus on the development of more potent and selective compounds with improved pharmacokinetic properties, and they should explore novel applications of pyridazinones in different unexplored therapeutic areas. The continued exploration and optimization of this privileged scaffold promise to yield innovative solutions to address unmet challenges in human health.

## Figures and Tables

**Table 1:** Anti-inflammatory activity of compounds **2**–**8** in comparison to ASA [16,17,18].

Compd.	mg/Kg	Carrageenin Rat Paw Edema Inhibition %	Compd.	mg/Kg	Carrageenin Rat Paw Edema Inhibition %
**2**	50 100	22 36	**6**	50 100	38 49
**3**	50 100	34 42	**7**	- 100	- 41
**4**	50 100	20 33	**8**	- 100	- 39
**5**	50 100	41 49	**ASA**	50 100	31 43

**Table 2:** Cardiovascular-related properties of compounds **9**–**29** and **33**–**48**.

Gen. Formula/Ref.	Compd.	R, R_1_, n, 4-4a Bond	Biological Activity (In Vivo/In Vitro)
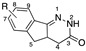 [23,24]	**9**	R = 7-NH_2_	antihypertensive, antithrombotic, antiaggregating, anti-inflammatory activity
	**10**	R = 7-NHCOCH_3_	antihypertensive, antithrombotic, antiaggregating, anti-inflammatory, antiulcer activity
	**11**	8-NO_2_	antithrombotic activity
	**12**	8-NH_2_	antithrombotic activity
	**13**	8-NHCOCH_3_	antithrombotic activity
	**14**	9-NHCOCH_3_	antithrombotic activity
	**15**	9-NH_2_	data not available
	**16**	7-OCH_3_	data not available
	**17**	7-OH	antithrombotic activity
	**18**	7-NHCH_3_	antihypertensive, antithrombotic activity
	**19**	7-N(CH_3_)COCH_3_	antihypertensive, antithrombotic activity
	**20**	7-N(CH_3_)COCH_2_CH_3_	antihypertensive, antithrombotic activity
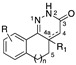 [25,26]	**21**	n = 1, R = 8-NH_2_, R_1_ = H, 4-4a single bond	antihypertensive, inotropic, antithrombotic activity
	**22**	n = 1, R = 8-NHCOCH_3_, R_1_ = H, 4-4a single bond	antihypertensive, inotropic, antithrombotic activity
	**23**	n = 1, R = 8-NHCOCH_3_, 4-4a double bond	antihypertensive, inotropic, antithrombotic activity
	**24**	n =2, R = 9-NH_2_, R_1_ = H, 4-4a single bond	antithrombotic activity
	**25**	n = 2, R = 9-NHCOCH_3_, R_1_ = H, 4-4a single bond	antithrombotic activity
	**26**	n =1, R = H, R_1_ = CH_2_OH, 4-4a single bond	antihypertensive activity
	**27**	n = 1, R = 8-OCH_3_, R_1_ = CH_2_OH, 4-4a single bond	antihypertensive, antithrombotic activity
	**28**	n = 1, R = 9-OCH_3_, R_1_ = CH_2_OH, 4-4a single bond	antithrombotic activity
	**29**	n = 1, R = H, R_1_ = CH_3_, 4-4a single bond	antithrombotic activity
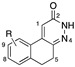 [29]	**33**	R = 9-NH_2_	antiaggregating, hypotensive, antihypertensive activity
	**34**	R = 9-NHCOCH_3_	antiaggregating, hypotensive, antihypertensive activity
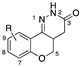 [32]	**35**	R = H	antiulcer activity
	**36**	R = 8-NH_2_	antithrombotic activity
	**37**	R = 8-NHCOCH_3_	antihypertensive, antithrombotic activity
	**38**	R = 9-OCH_3_	antiulcer activity
	**39**	R = 8,9-OCH_3_	antiulcer activity
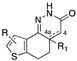 [33,34]	**40**	R = R_1_ = H, 4-4a single bond	antithrombotic activity
	**41**	R = 8-NHCOCH_3_, R_1_ = H, 4-4a single bond	antithrombotic activity
	**42**	R = 9-NHCOCH_3_, R_1_ = H, 4-4a single bond	hypotensive activity
	**43**	R = 9-NHCOCH_3_, 4-4a double bond	hypotensive activity, antiaggregating activity
	**46**	R = H, R_1_ = CH_3_ 4-4a single bond	antihypertensive, antiaggregating activity
	**47**	R = 8-NHCOCH_3_, R_1_ = CH_3_, 4-4a single bond	hypotensive, antiaggregating activity
	**48**	R = 9-NHCOCH_3_, R_1_ = CH_3_, 4-4a single bond	hypotensive, antiaggregating activity
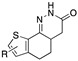 [33]	**44**	R = 7-NHCOCH_3_	hypotensive, antiaggregating activity
	**45**	R = 8-NHCOCH_3_	hypotensive, antihypertensive activity, antiaggregating activity

**Table 3:** Antiulcer properties of compounds **55**–**63**.

Gen. Formula/Ref.	Compd.	X, R_1_, 4-4a Bond	R	Biological Activity (In Vivo)
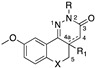 [37]	**55**	X = O, 4-4a double bond	H	antiulcer activity (ethanol and ASA model), antisecretory activity
	**56**	X = CH_2_, 4-4a double bond	H	antiulcer activity (ethanol model)
	**57**	X = CH_2_, R_1_ = CH_3_, 4-4a single bond	H	antiulcer activity (ethanol model)
	**58**	X = O, R_1_ = H, 4-4a single bond	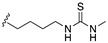	antiulcer activity (ethanol model)
	**59**	X = O, 4-4a double bond	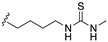	antiulcer activity (ethanol and ASA model)
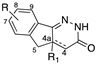 [38]	**60**	4-4a double bond	7,8-(OCH_3_)_2_	antiulcer activity (ethanol and indomethacine model), antisecretory activity
	**61**	4-4a double bond	6,9-(OCH_3_)_2_	antiulcer activity (ethanol and indomethacine model)
	**62**	R_1_ = H, 4-4a single bond	7,8-(OCH_3_)_2_	antiulcer activity (ethanol and indomethacine model)
	**63**	R_1_ = H, 4-4a single bond	6,9-(OCH_3_)_2_	antiulcer activity (ethanol and indomethacine model)

**Table 4:** Anticancer properties of selected tricyclic pyridazinones.

Compd./Ref.	Human Cancer Cell Line/Target	Compd./Ref.	Human Cancer Cell Line/Target
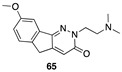 [52]	colon (LoVo and LoVo/DX), murine leukemia (L1210 and L1210/CDDP)	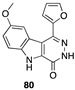 [65]	hepatocellular (Hub-7), colorectal (Caco2), breast (MDA-MB-231) DYRKIA inhibitor
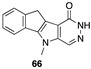 [53]	renal (ACHN), leukemia (MOLT-4), non-small-cell lung (NCI-H460), colon (HCT-116), CNS (SF-295)	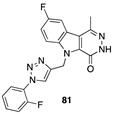 [66]	breast (MDA-MB-231, MCF-7), CNS (U-87, IMR-32) PI3K inhibitor (in silico study).
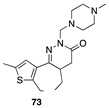 [55]	non-small-cell lung (EKVX, HOP-95), CNS (SNB-75)	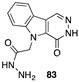 [67]	breast (MCF-7) PI3K inhibitor (in silico study).
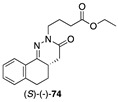 [57]	STAT3 inhibitor	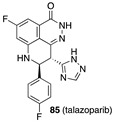 [70]	PARP-1 inhibitor clinical use: breast cancer
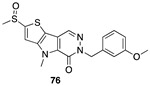 [59]	PKM2 activator	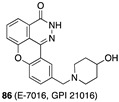 [71]	PARP-1 inhibitor clinical use: melanoma
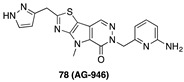 [60]	PKR activator	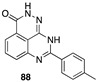 [72]	PARP-1 inhibitor
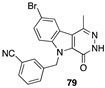 [61]	hepatocellular (Hub-7), colorectal (Caco2, HCT-116), breast (MDA-MB-231), prostate (PC3), lung (NCI-H727). PI3Kα inhibitor	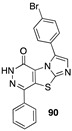 [74]	leukemia (HL-60TB, K-562, RPMI-8226), colon (HCT-116, HT29), melanoma (MDA-MB-435), ovarian (OVCAR-3), breast (MDA-MB468) DHFR inhibitor

**Table 5:** ALR2 inhibition data of compounds **96**–**107**.

(Gen.) Formula/Ref.	Compd.	R	R_1_	ALR2 IC_50_ μM or (% Inhibn.)
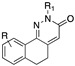 [81,82]	**96**	H	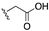	12.6
	**97**	7,9-(CH_3_)_2_	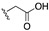	(31, 48 μM)
	**98**	H	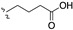	11.4
	**99**	8,9-(OCH_3_)_2_	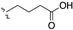	(35, 79 μM)
	**100**	7,9-(CH_3_)_2_	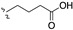	17.4
	**101**	7-OCH_3_	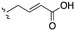	4.25
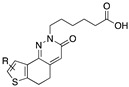 [84]	**103**	H		7.6
	**104**	8-CH_3_		18.0
	**105**	8-Cl		31.4
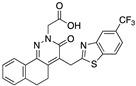 [83]	**102**			0.15
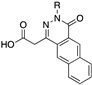 [85]	**106**	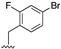		0.42
	**107**	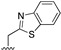		0.046

**Table 6:** BZR-binding affinity of compounds **109**–**113**.

(Gen.) Formula/Ref.	Compd.	X, n, R, R_1_	[^3^H]Diazepam K_i_ (nM)
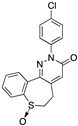 [87]	**109** (Y-23684)		42.0
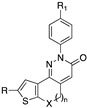 [87,88]	**110**	X = SO_2_, n = 2, R = CH_2_CH_3_, R_1_ = Cl	1.1
	**111**	X = SO_2_, n = 2, R = Br, R_1_ = Cl	0.61
	**112**	X = CH_2_, n = 1, R = CH(OH)CH_3_, R_1_ = CH_3_	6.4
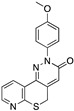 [89]	**113**		695.0 ([^3^H]Flumazenil)

**Table 7:** Biological properties of representative tricyclic pyridazinones previously described.

Compd./Ref.	Target	Compd./Ref.	Target
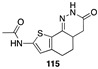 [95]	MMP-8 inhibitor	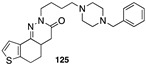 [106]	cholinesterase (AChE, BChE) inhibitor
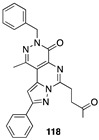 [98]	PDE5 inhibitor	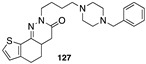 [106]	cholinesterase (AChE, BChE) inhibitor
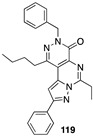 [101]	hA1 antagonist	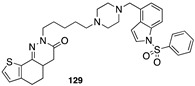 [106]	AChE inhibitor, 5-HT_6_
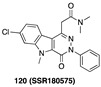 [103]	TSPO	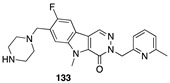 [108]	PKM2 activator
